# Characterization of a *G. max* × *G. soja* nested association mapping population and identification of loci controlling seed composition traits from wild soybean

**DOI:** 10.1007/s00122-025-04848-5

**Published:** 2025-03-07

**Authors:** Linfeng Chen, Earl Taliercio, Zenglu Li, Rouf Mian, Thomas E. Carter, He Wei, Chuck Quigely, Susan Araya, Ruifeng He, Qijian Song

**Affiliations:** 1 Present Address: College of Agriculture, Henan University of Science and Technology, Luoyang, Henan Province China; 2 Soybean Genomics and Improvement Laboratory, Beltsville Agricultural Research Center, USDA-ARS, Beltsville, MD USA; 3 Soybean and Nitrogen Fixation Research Unit, USDA-ARS, Raleigh, NC USA; 4 Department of Crop and Soil Sciences, Institute of Plant Breeding, Genetics, and Genomics, University of Georgia, Athens, GA USA; 5 Present Address: Institute of Crop Molecular Breeding, Henan Academy of Agricultural Sciences, Zhengzhou, Henan Province China

## Abstract

Wild soybean (*Glycine soja* Siebold & Zucc.) has valuable genetic diversity for improved disease resistance, stress tolerance, seed protein content and seed sulfur-containing amino acid concentrations. Many studies have reported loci controlling seed composition traits based on cultivated soybean populations, but wild soybean has been largely overlooked. In this study, a nested association mapping (NAM) population consisting of 10 families and 1107 recombinant inbred lines was developed by crossing 10 wild accessions with the common cultivar NC-Raleigh. Seed composition of the F_6_ generation grown at two locations was phenotyped, and genetic markers were identified for each line. The average number of recombination events in the wild soybean-derived population was significantly higher than that in the cultivated soybean-derived population, which resulted in a higher resolution for QTL mapping. Segregation bias in almost all NAM families was significantly biased toward the alleles of the wild soybean parent. Through single-family linkage mapping and association analysis of the entire NAM population, new QTLs with positive allele effects were identified from wild parents, including 5, 6, 18, 9, 16, 17 and 20 for protein content, oil content, total protein and oil content, methionine content, cysteine content, lysine content and threonine content, respectively. Candidate genes associated with these traits were identified based on gene annotations and gene expression levels in different tissues. This is the first study to reveal the genetic characteristics of wild soybean-derived populations, landscapes and the extent of effects of QTLs and candidate genes controlling traits from different wild soybean parents.

**Supplementary Information:**

The online version contains supplementary material available at 10.1007/s00122-025-04848-5.

## Introduction

Soybean (*Glycine max* (L.) Merr.) is an important leguminous crop that provides protein and oil for human food, animal feed or industrial products. Soybean meal, a by-product from soybean oil extraction process, is increasingly important as animal feed (Pettersson and Pontoppidan 2013), with approximately 77% of soybean meal used in the animal feed industry as a source of protein and amino acids (Kerley and Allee. 2003).

Soybean seed protein contains all of the amino acids consumed by human and animals, but is relatively low in the essential sulfur-containing amino acids (cysteine, methionine), lysine and threonine, which are essential for monogastric animals because these animals cannot synthesize the amino acids and therefore must get them from their feed (George and De Lumen 1991). Supplementing soybean meal with these amino acids, especially cysteine and methionine, in the animal diet adds cost to the producer and may lead to leaching during soybean meal processing and the formation of undesirable volatile sulfides after bacterial degradation (Warrington et al. 2015). The development of soybean cultivars with enhanced amino acids balance would increase economic value and reduce negative environmental impact.

The domestication of soybean from the wild soybean was followed by centuries of selection, and in the past 70 years, intensive breeding and selection for higher seed yield have resulted in dramatically reduced genetic variability of modern US soybean cultivars (Hyten et al. 2006). Wild soybean is the progenitor of cultivated soybean (Hymowitz and Newell 1981; Hymowitz 1970) and has not gone through the bottleneck of having genes selected for agriculture, and is much more diverse than its cultivated counterparts (Li et al. 2010; Hyten et al. 2006). It can also be hybridized with cultivated soybean lines without the need for embryo rescue, tissue culture and other means. Wild soybean has many interesting traits like disease and abiotic-stress resistance (Tuyen et al. 2010; Diers et al. 1992; Sun et al. 1990), and is a valuable genetic resource for increasing the seed protein content and seed sulfur-containing amino acid concentration in elite soybean. For example, according to our analysis of 993 wild soybean and 16,126 cultivated soybeans reported in GRIN, the average seed protein concentration of wild soybean was 46.8% and that of cultivated soybean was 43.0%, and the average cysteine and methionine contents were  higher in wild soybean than cultivated soybean. The concentration range of the above seed composition in wild soybean was also higher than that of the cultivated soybean (La et al. 2019). Wild soybean is not just a source of diversity and desired traits, breeders have successfully used it in plant breeding and released high-yield, high-protein and good agronomic performance lines (Eickholt et al. 2019; Taliercio et al. 2023; Fallen et al. 2024).

Since 1992, a total of 51 studies have reported QTLs for protein and oil concentration in soybean, and more than 240 QTLs controlling seed protein and oil content have been documented, and a total of three studies have reported seven QTLs for cysteine and methionine concentration recorded at SoyBase (https://www.soybase.org/). However, of the 51 studies, only few were related to wild soybean and only one wild soybean accession and one parent with 25% of wild soybean in its pedigree were involved (Diers et al. 1992; Sebolt et al. 2000; Nichols et al. 2006; Wang et al. 2004; Brummer et al. 1997). In the soybean *G. max* (A81-356,022) × PI 468916 population, two major QTL controlling seed protein and oil concentration from wild soybean were reported on chromosome 20 and chromosome 15 (Diers et al. 1992). Subsequently, the lines from the same population were backcrossed for the estimation of QTL effect on yield and other traits (Nichols et al. 2006; Sebolt et al. 2000). Fine mapping of the QTL on chromosome 20 was also attempted (Nichols et al. 2006). In another cross (M82-806 × HHP) with a 25% *G. soja* pedigree, the two high-protein QTL on chromosomes 15 and 20 were confirmed (Brummer et al. 1997). The genes associated with the high-protein QTL on chromosome 20 and chromosome 15 were recently cloned (Fliege et al. 2022; Goettel et al. 2022; Zhang et al. 2020). At present, there are no reports on the QTL of sulfur-containing amino acids in wild soybean progeny though progeny with elevated sulfur-containing amino acids has been reported (Eickholt et al. 2019).

Soybean protein and oil contents are complex quantitative traits controlled by many genes and affected by the interaction between genotype and environment. Previous studies have shown a significant negative correlation between soybean protein and oil contents (Hwang et al. 2014; Warrington et al. 2015; Lee et al. 2019) caused by either inversely pleiotropic effects or tight linkage (Chung et al. 2003). Although dissecting the genetic basis of soybean protein and oil contents will facilitate recombining loci to reduce negative correlation in cultivars, finding loci that control total oil and protein content rather than just protein or oil content individually, may help breed lines with high total protein and oil content.

Nested association mapping (NAM) populations are developed by crossing multiple representative founders to a common parent, followed by generations of selfing in each family. NAM takes advantages of both linkage mapping and associated mapping to improve statistical power and mapping resolution while decreasing confounding population structure (Yu et al. 2008). Since the first maize NAM population publicly released in 2009, many NAM populations have been developed in various crops, such as rice (Fragoso et al. 2017), wheat (Kidane et al. 2019), sorghum (Bouchet et al. 2017), barley (Maurer et al. 2015) and rapeseed (Hu et al. 2018). A cultivated soybean NAM population consisting of 5600 RILs from 40 families was also created (Diers et al. 2018; Song et al. 2017). Genetic association analysis of the population resulted in the identification of 107 marker-trait associations (MTAs) for the content of seed protein, oil and meal protein (Diers et al. 2023). The population was useful to detect and fine-map QTL controlling complex quantitative traits.

The objectives of this study were to create a NAM population derived from wild and cultivated soybean germplasm, characterize the population and identify genomic loci controlling protein content, oil content, total protein and oil content, and essential amino acid contents, specifically from wild soybean.

## Materials and methods

### Creation of *G. max* × *G. soja* NAM population

A NAM population consisted of 10 RIL families was developed by crossing 10 diverse wild soybean accessions (*G. soja*) to a common soybean (*G. max*) cultivar “NC-Raleigh” (Burton et al. 2006) (Table 1). The 10 wild soybean parents were selected from a diverse wild soybean group consisting of germplasm from different countries with a range of protein content and oil content. The seeds from the 10 crosses were advanced to the F_6_ generation using a single seed descent (SSD) method (Brim 1966). A total of 1107 RILs were obtained, with approximately 110 RILs from each family. The parents and NAM RILs of the 10 crosses were grown in fields at Beltsville, MD, and Clayton, NC over 2 years (2018 and 2019). Field tests were conducted using a randomized complete block design with two replicates of hill plots at Beltsville, and complete randomized design with one replication at Clayton, NC each year.

**Table 1:** List of the 10 crosses between NC-Raleigh (MG VII) and a diverse set of *G. soja* accessions

Code	Crosses	Wild soybean maturity group	Origin of wild parent	Code	Crosses	Wild soybean maturity group	Origin of wild parent
NAM01	NC-Raleigh × PI378684B	VI	Japan	NAM06	NC-Raleigh × PI424007	V	South Korea
NAM02	NC-Raleigh × PI378690	VII	Japan	NAM07	NC-Raleigh × PI424045	V	South Korea
NAM03	NC-Raleigh × PI378696B	VI	Japan	NAM08	NC-Raleigh × PI424083A	V	South Korea
NAM04	NC-Raleigh × PI407020	V	Japan	NAM09	NC-Raleigh × PI549032	III	China
NAM05	NC-Raleigh × PI407228	V	South Korea	NAM10	NC-Raleigh × PI562551	V	South Korea

### Seed composition measurements

For an analysis of seed composition, whole soybean seeds were ground into powder and then analyzed on a DA 7250 NIR Analyzer at the University of Georgia. The calibration equation for the DA 7250 NIR analyzer was provided by the manufacturer and was developed using the Association of Official Seed Certifying Agency (AOSCA) approved method for HPLC amino acid analysis (Warrington et al. 2015). NIR measurements of soybean amino acids have been commonly used by different soybean research laboratories for QTL mapping purposes (Khandaker et al. 2015; Panthee et al. 2006a; Wang et al. 2015; Warrington et al. 2015). The protein content, oil content and amino acid contents were reported in g kg^−1^ on a moisture-free basis and then calculated as the relative percentage of seed dry-weight. The total protein and oil content was calculated by adding the contents of protein and oil.

### Genotyping and genetic map construction

Genomic DNA was extracted from young leaves of RILs and 11 parents using the cetyltrimethylammonium bromide (CTAB) method (Murray and Thompson 1980). The 11 NAM parents were genotyped with the SoySNP50K BeadChip assay (Song et al. 2013). The 1107 RILs derived from the 10 NAM families were genotyped with BARCSoySNP6K BeadChip assay (Song et al. 2020) and then were imputed to high density markers based on the SoySNP50K markers of the11 parents using AlphaPlantImpute software that showed higher imputation accuracy for biparental populations than other software (Chen et al. 2022). The linkage map position of the markers was inferred according to the method described previously (Chen et al. 2022). The maps were subsequently used for SNP QTL linkage mapping.

### Phenotypic data analysis

The descriptive statistical analysis on the protein, oil and amino acid contents of the NAM population was conducted using SAS 9.4, including the maximum, minimum, average, standard deviation and coefficient of variation (CV). The analysis of variance (ANOVA) was performed using the PROC GLM of SAS 9.4 with location, replication within locations and genotype × location as random effects. The model for the phenotype can be expressed as *y*_*ijk*_ = *µ* + *G*_*i*_ + *E*_*j*_ + *GE*_*ij*_ + *R*_*k(j)*_ + *e*_*ijk*_, where *µ* is the population mean, *G*_*i*_ is the effect of the *i-*th genotype, *E*_*j*_ is the effect of the *j-*th location, *GE*_*ij*_ is the interaction effect between the *i-*th genotype and the *j-*th location, *R*_*k(j)*_ is the effect of the *k-*th block within the *j-*th location, and *e*_*ijk*_ is the random error. The broad-sense heritability (*H*) of all the traits was calculated as *H* = *σ*^*2*^_*g*_*/* (*σ*^*2*^_*g*_ + *σ*^*2*^_*ge*_*/n* + *σ*^*2*^_*e*_*/nr*), where *σ*^*2*^_*g*_^*2*^is the genotypic variance, *σ*^*2*^_*ge*_ is the interaction variance of genotype × environment, *σ*_*e*_ is the error variance, *n* is the number of locations, and *r* is the number of replications within each location. To minimize the effects of environmental variance, the best linear unbiased prediction (BLUP) values were calculated using R software package “*lme4*” (Merk et al. 2012) for each trait based on the data collected from different environments. Phenotypic correlation analysis was performed using R software package “*ggcor*.”

### Linkage disequilibrium, population structure and principal component analyses

Based on the imputed dataset, linkage disequilibrium (LD) was measured by the squared correlation coefficient *r*^*2*^ of pairwise SNPs using popLDdecay (Zhang et al. 2019), where the maximum intervals between pairwise SNPs were set to 1500 kb. The LD decay was calculated as the physical distance at which the average pairwise *r*^*2*^ dropped to the half of its maximum value. The principal component analysis (PCA) for all the RILs and parents of NAM population was conducted using PLINK. The first two eigenvectors were selected to show the relationship between lines, the plots of lines were then visualized using R software package “*ggplot2*”. The population structure for all the families of NAM population was estimated using ADMIXTURE software.

### Recombination events and segregation distortion

The number of recombination events (REs) was counted based on the number of allele pattern changes along the 20 chromosomes of each RIL. The details of REs calculation were previously described by Song et al. (2017). Segregation distortion (SD) refers to the deviation between the observed allelic ratio at a locus and the expected Mendelian ratio in a biparental segregation population. The proportion of alleles at each locus was tested for distortion against approximate 0.48 (homozygote 1):0.03 (heterozygote):0.48 (homozygote 2) segregation ratio of each marker within a given RIL family using chi-square test. A threshold of *P* < 0.01 was used to determine the significance of the SD. The euchromatic and heterochromatic regions in soybean whole genome sequence were defined in previous study (Song et al. 2016) and were used to compare the SD differences between these two regions.

### QTL linkage mapping

Separate linkage mapping (SLM) of soybean protein, oil and amino acid contents in each NAM family was performed using ICIMapping 4.2 (Meng et al. 2015). The SNPs were filtered to remove markers missing > 0.2 in each NAM family before QTL mapping. The genotypic data were recorded as 0, 1, 2, − 1, for non-common wild parental homozygous, heterozygous, common parental homozygous alleles and missing genotypes, respectively. The ICIM method in ICIMapping 4.2 software was performed with the scanning step of 1 cM and probability of 0.001. The logarithm of odds (LOD) value was determined by 1000 permutation tests with type I error *α* = 0.05.

### The restricted two-stage multi-locus genome-wide association study

The restricted two-stage multi-locus (RTM) genome-wide association study (GWAS) (He et al. 2017) was used to identify QTLs associated with soybean seed protein, oil and amino acid contents. The SNPs within a linkage disequilibrium block were grouped and termed SNPLDB markers based on imputed dataset using built-in program in RTM-GWAS software. According to the internal software algorithm, the comprehensive population structure was inferred by genetic similarity coefficient (GSC) matrix based on SNPLDBs, in which the top 10 eigenvalues of the GSC were used to correct the population structure deviation. The RTM-GWAS procedures were carried out in two stages. At the first stage, a single-locus association analysis was performed based on a simple linear model to initially screen the SNPLDB markers, at the second stage, the significant loci obtained in the first stage were screened by stepwise regression with forward selection and backward elimination based on a multi-locus model to identify genome-wide QTLs, and to estimate the allelic effect value. The significance level was set at 0.05 for the initial screening of markers and the multi-locus stepwise regression association analysis.

### Candidate gene annotation and expression analysis

The candidate genes of soybean protein, oil and amino acid contents were inferred according to the gene annotation information of the soybean reference genome Wm82.v2.a1 and QTL position. The genes within the physical range of the associated SNPLDBs (± 100 kb) were retrieved. According to the functional annotations downloaded from the SoyBase (http://www.soybase.org) and the functional annotation of *Arabidopsis* orthologs, the retrieved genes with functions related to the studied traits were considered as candidate genes. The gene expression levels at different tissues were obtained from public domain SoyOmics at https://ngdc.cncb.ac.cn/soyomics/expression_tool/ (Liu et at. 2023). Raw FPKM data were transformed log_2_ to plot expression heatmaps, and the SoyOmics tool used tspex (tissue-specificity calculator) to determine tissue specificity of genes.

## Results

### Population structure and LD analysis

**Fig. 1:**
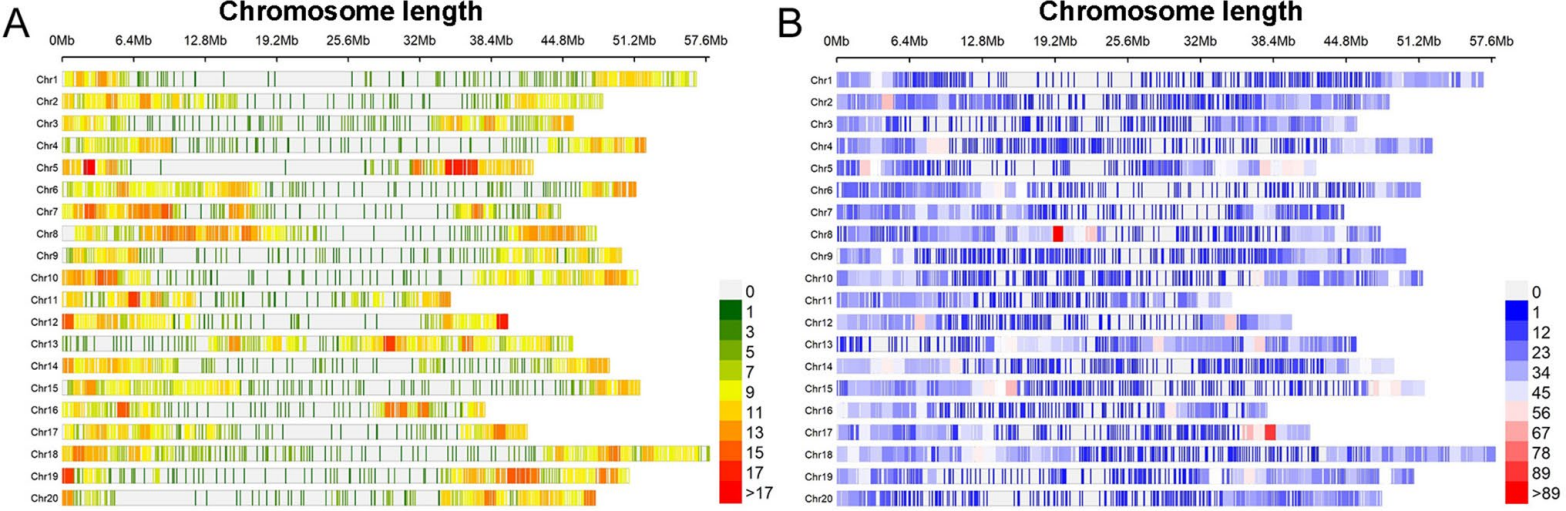
SNP densities per Mb in the genome based on the number of SNPs in BARCSoySNP6K assay (**A**) and the number of SNP imputed using parental SoySNP50K dataset (**B**)

**Fig. 2:**
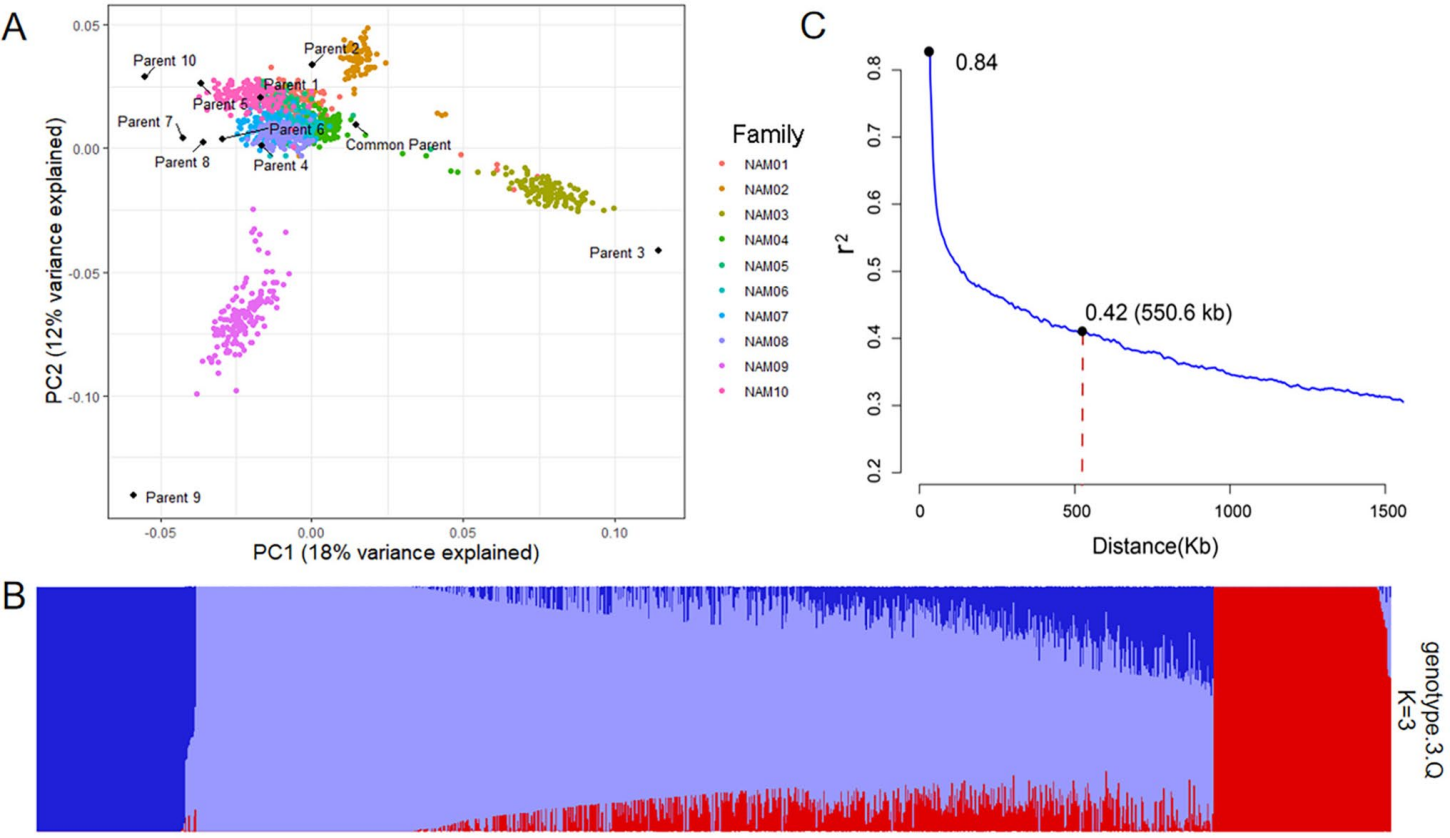
Analysis of population structure and linkage disequilibrium (LD) for the soybean NAM population. **A** Principal component analysis (PCA) plot of the NAM parents and RILs based on the first two principal components. Different color dots represent different families. Each dot represents a recombinant inbred line (RIL). **B** Population structure of the NAM population revealed by STRUCTURE. The NAM population was divided into three groups based on the proportion of membership. **C** Genome-wide LD decay. The LD decay was calculated based on the average *r*^2^ value of markers within a 1500 kb window

After filtering, a total of 5049 SNPs were successfully genotyped for 1107 RILs (Fig. 1A). After imputation with the parental SNPs from SoySNP50K assay, a total of 17 K SNPs was obtained per line (Fig. 1B). Principal component analysis (PCA) separated all the offspring of NAM population into three main clusters surrounding the common parent (Fig. 2A), which corresponded to the three groups revealed by structure analysis (Fig. 2B). PCA1 and PCA2 explained 18% and 12% of the genetic variance, respectively, suggesting a weak stratification within the NAM population. The LD decay was estimated at 550.6 kb (*r*^2^ = 0.42) (Fig. 2C).

### Recombination events of the NAM population

Recombination events (REs) were determined in each family based on the polymorphic markers. A total of 120,477 REs was identified among the 1107 RILs in the NAM population with an average of 111.0 (standard deviation: 25) per RIL (Table 2), significantly higher than the previously reported RE of 58.5 for the NAM population with only cultivated soybean. The number of REs for the majority of the RILs was quite consistent, 89.6% (922) of the RILs contained less than 200 REs. Of the 120,477 REs, a total of 25,467 (21.1%) were unique, i.e., these REs only occurred in one RIL within a family, and 95,010 (78.9%) overlapped in at least two RILs within a family. The average number of unique REs per RIL was 23.9 among the 10 NAM families.

**Table 2:** Number of RILs, polymorphic loci and recombination events (REs) in each NAM family

Population ID	No. of RILs remaining after quality control	No. of polymorphic loci from SoySNP6K	Total number of REs	Average number of REs per RIL	No. of unique REs among RILs	Proportion of total unique REs among RILs in each family	No. of REs occurring in at least two RILs in each family	No. of unique REs per RIL in each family
NAM01	97	2,503	10,364	106.85	2,406	0.23	7,958	25
NAM02	76	2,459	9,935	130.72	2,392	0.24	7,543	31
NAM03	114	2,628	12,130	106.40	2,557	0.21	9,573	22
NAM04	113	2,195	11,543	102.15	2,123	0.18	9,420	19
NAM05	85	2,789	8,274	97.34	2,583	0.31	5,691	30
NAM06	136	2,271	15,229	111.98	2,250	0.15	12,979	17
NAM07	103	2,899	11,511	111.76	2,790	0.24	8,721	27
NAM08	101	2,782	17,223	170.52	2,730	0.16	14,493	27
NAM09	145	2,930	13,195	91.00	2,845	0.22	10,350	20
NAM10	137	2,956	11,073	80.82	2,791	0.25	8,282	20
Total	1,107	26,412	120,477	1,109.55	25,467	/	95,010	239
Average	110.7	2,641.2	12,047.7	110.95	2,546.7	0.22	9,501	23.9

### Segregation distortion of SNPs among families

SD is an important factor influencing the linkage mapping in biparental populations. Of the 26,412 polymorphic loci observed in the 10 families, a total of 1892 (7.16%) SNPs exhibited SD at *P* < 0.01 (Table 3) and 42.71% of the SD SNPs were in different LD blocks. The average percentages of SNPs with SD in euchromatic and heterochromatic regions across families were 7.33% and 7.25%, respectively. The percentage of SNPs with SD in 10 RIL families varied widely from 2.50% for NAM10 to 15.89% for NAM08. Among the 10 families, only NAM03 had more SNPs with SD favoring common parent NC-Raleigh alleles, while the other nine families had more SNPs with SD favoring wild soybean parental alleles. Among all SNPs with SD, 80.07% of the SNPs favored wild soybean parental alleles, and 19.93% of the SNPs favored common parental alleles, it indicated that some alleles of wild parents had advantages over the alleles from cultivated soybean.

**Table 3:** Number of polymorphic loci and segregation distortion (SD) loci in the euchromatic and heterochromatic regions in each family

Family	No. of polymorphic loci in the euchromatic regions	No. of polymorphic loci in the heterochromatic regions	No. of SNPs with SD in the euchromatic regions	No. of SNPs with SD in the heterochromatic regions	Percentage of SNPs with SD in euchromatic regions (%)	Percentage of SNPs with SD in heterochromatic regions (%)	Percentage of SNPs with SD in euchromatic and heterochromatic regions (%)	No. of SNPs with SD that favor wild soybean parental alleles	No. of SNPs with SD that favor NC-Raleigh alleles	Percentage of SNPs with SD located in LD blocks (%)
NAM01	2,071	432	115	42	5.55	9.72	6.27	93	64	27.39
NAM02	2,045	414	60	7	2.93	1.69	2.72	57	10	37.31
NAM03	2,155	473	104	11	4.83	2.33	4.38	49	66	39.13
NAM04	1,819	376	172	44	9.46	11.70	9.84	199	17	35.19
NAM05	2,316	473	73	6	3.15	1.27	2.83	50	29	50.63
NAM06	1,865	406	280	56	15.01	13.79	14.80	295	41	24.11
NAM07	2,393	506	189	31	7.90	6.13	7.59	190	30	50.00
NAM08	2,287	495	366	76	16.00	15.35	15.89	401	41	39.82
NAM09	2,407	523	145	41	6.02	7.84	6.35	122	64	50.54
NAM10	2,430	526	60	14	2.47	2.66	2.50	59	15	72.97
Total	21,788	4,624	1,564	328	/	/	/	1,515	377	/
Mean	2,178.8	462.4	156.4	32.8	7.33	7.25	7.32	151.5	37.7	42.71

### Residual heterozygosity in the genome

Residual heterozygosity in RIL population is excellent genetic resource for rapid and fine mapping of QTLs in the heterozygous regions. The residual heterozygosity (RH) of the F_6_ RILs in each family varied from 2.03% in NAM09 to 3.06% in NAM10, with an average of 2.42% in all NAM families. This was slightly lower than the expected rate of 3.13% for the F_6_ plants. In addition, the RH of the F_6_ RILs was averaged 2.41% in the euchromatic regions and 2.43% in the heterochromatic regions, which was not significantly different (Table 4). The correlation of the number of RH between the two regions across the RIL families was 0.80, which was significantly and positively correlated. The residual heterozygous loci for all the RILs in the NAM population covered the whole genome (Fig. S1), indicating that the NAM population is an important genetic resource for subsequent fine mapping of QTL in the population from target residual heterozygous lines.

**Table 4:** Number and percentage of residual heterozygotes in euchromatic and heterochromatic regions by family

Family	Euchromatic regions				Heterochromatic regions			
	No. of homozygous SNPs with wild soybean alleles	No. of heterozygous SNPs	No. of homozygous SNPs with NC-Raleigh alleles	Percentage of heterozygous SNPs	No. of homozygous SNPs with wild soybean alleles	No. of heterozygous SNPs	No. of homozygous SNPs with NC-Raleigh alleles	Percentage of heterozygous SNPs
NAM01	103,823	9,654	264,812	2.55	22,855	2,004	55,017	2.51
NAM02	78,518	6,331	210,372	2.14	14,899	1,240	46,053	1.99
NAM03	116,934	11,132	320,955	2.48	25,304	2,712	65,601	2.90
NAM04	119,527	10,604	321,519	2.35	24,190	2,393	67,707	2.54
NAM05	92,490	8,373	232,323	2.51	18,902	1,508	49,979	2.14
NAM06	133,624	13,134	375,396	2.52	27,527	2,808	79,600	2.55
NAM07	115,573	9,165	281,237	2.26	24,437	1,858	59,257	2.17
NAM08	114,345	9,475	277,102	2.36	24,062	1,917	58,179	2.28
NAM09	165,216	11,184	396,973	1.95	36,014	2,540	81,648	2.11
NAM10	155,460	16,387	375,908	2.99	32,991	3,630	79,230	3.13
Total	1,195,510	105,439	3,056,597	/	251,181	22,610	642,271	/
Mean	119,551	10,543.9	305,659.7	2.41	25,118.1	2,261	64,227.1	2.43

### Phenotypic variation and correlation analysis

**Fig. 3:**
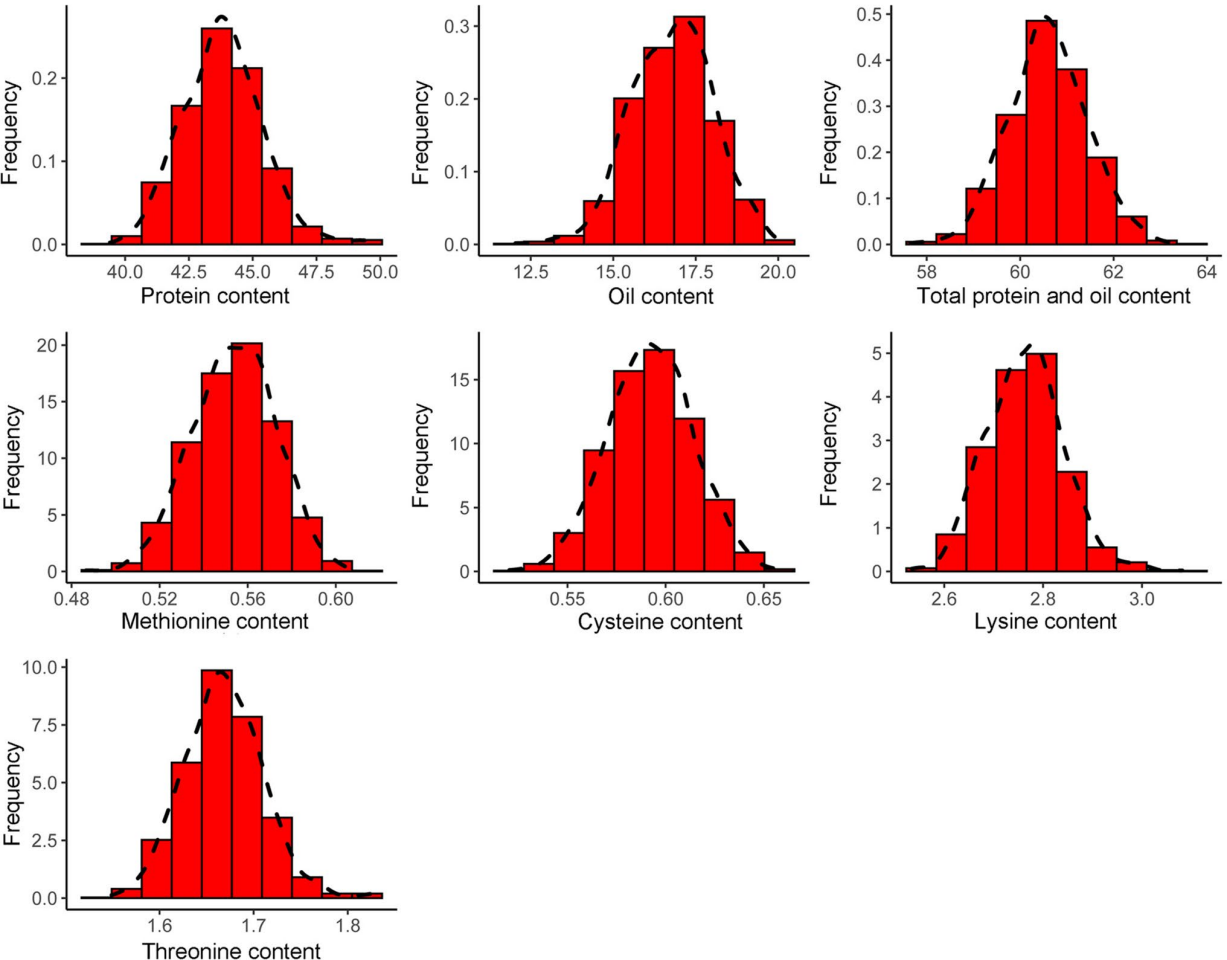
Trait phenotypic distribution based on scaled best liner unbiased predictor (BLUP) values in all environments of the NAM population

**Fig. 4:**
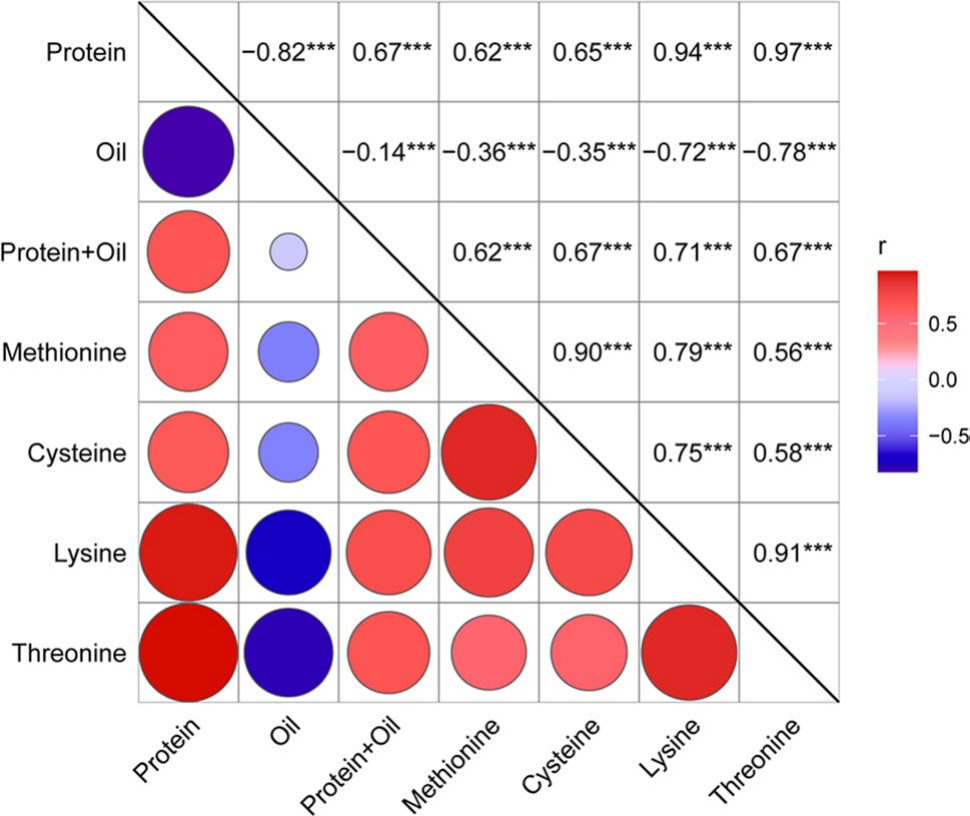
Phenotypic correlation among seven traits based on scaled BLUP values from 1107 RILs in the NAM population. ***significant at *P* < 0.001

Across the four environments, the NAM population varied widely in seed protein (37.12–51.92%), oil (11.6–21.67%), total protein and oil (56.47–64.80%), methionine (0.47–0.65%), cysteine (0.47%-0.72%), lysine (2.42–3.16%) and threonine (1.45–1.90%) (Fig. 3). ANOVA showed that all traits were significantly affected by the genotype and environment (*P* < 0.001). The *h*^*2*^ values of all traits exceeded 85%, indicating that these traits were stable and less affected by environmental factors (Table 5). Consistent with numerous previous studies, there was a strong negative correlation between protein and oil contents (*r* = − 0.82, *P* < 0.001). In addition, seed protein content was positively correlated with four amino acid contents (methionine* r* = 0.62, cysteine* r* = 0.65, lysine* r* = 0.94, threonine* r* = 0.97; *P* < 0.001), while oil content was negatively correlated with each amino acid content (methionine* r* = − 0.36, cysteine *r* = − 0.35, lysine *r* = − 0.72, threonine* r* = − 0.78; *P* < 0.001). The contents of the four amino acids were all significantly and positively correlated (*P* < 0.001) (Fig. 4).

**Table 5:** Descriptive statistics, ANOVA and *H* of all studied traits in the soybean NAM population

Trait	Environment		Mean (%)	SD^a^	Minimum (%)	Maximum (%)	CV^b^ (%)	Skewness	Kurtosis	F_*G*_^c^	F_*E*_^d^	F_*R(E)*_^e^	F_*G*×*E*_^f^	H (*%*)
Protein content	2018_Beltsville	43.53		2.47	35.99	52.34	5.67	0.07	0.17	12.79***	204.58***	170.68***	1.38***	92.42
	2018_Clayton	44.49		2.19	36.38	51.86	4.92	0.18	0.23					
	2019_Beltsville	43.45		1.84	38.15	51.42	4.24	0.35	0.68					
	2019_Clayton	43.81		1.94	37.94	52.04	4.43	0.07	0.30					
	Mean	43.82		2.11	37.12	51.92	4.82	0.17	0.35					
Oil content	2018_Beltsville	16.12		1.60	10.69	21.19	9.92	− 0.11	− 0.02	24.03***	1268.85***	163.91***	1.55***	95.38
	2018_Clayton	16.58		1.50	11.51	22.65	9.05	− 0.03	0.18					
	2019_Beltsville	17.22		1.43	11.79	21.37	8.28	− 0.20	0.20					
	2019_Clayton	17.28		1.45	12.57	21.48	8.37	− 0.06	− 0.15					
	Mean	16.80		1.50	11.64	21.67	8.91	− 0.10	0.05					
Protein + Oil content	2018_Beltsville	59.65		1.52	53.93	64.31	2.56	− 0.14	0.12	7.06***	957.27***	68.86***	1.19***	88.72
	2018_Clayton	61.06		1.39	56.79	65.58	2.27	− 0.05	0.09					
	2019_Beltsville	60.67		1.17	57.29	64.71	1.92	0.10	− 0.09					
	2019_Clayton	61.09		1.04	57.87	64.61	1.70	0.03	0.06					
	Mean	60.62		1.28	56.47	64.80	2.11	− 0.02	0.05					
Methionine content	2018_Beltsville	0.54		0.03	0.45	0.63	5.56	− 0.12	− 0.19	7.91***	344.93***	54.75***	1.32***	87.81
	2018_Clayton	0.56		0.03	0.47	0.66	5.23	0.04	− 0.23					
	2019_Beltsville	0.56		0.03	0.47	0.64	4.70	0.03	− 0.21					
	2019_Clayton	0.55		0.03	0.47	0.65	5.22	− 0.03	− 0.07					
	Mean	0.55		0.03	0.47	0.65	5.18	− 0.02	− 0.18					
Cysteine content	2018_Beltsville	0.59		0.40	0.40	0.71	6.54	− 0.06	0.29	5.43***	77.41***	66.68***	1.11**	86.02
	2018_Clayton	0.60		0.40	0.50	0.71	6.13	0.18	− 0.23					
	2019_Beltsville	0.59		0.30	0.50	0.72	5.51	0.17	− 0.12					
	2019_Clayton	0.59		0.30	0.48	0.74	5.90	0.11	0.34					
	Mean	0.59		0.35	0.47	0.72	6.02	0.10	0.07					
Lysine content	2018_Beltsville	2.76		0.12	2.35	3.20	4.49	− 0.02	0.14	11.07***	171.09***	154.37***	1.36***	91.42
	2018_Clayton	2.80		0.11	2.42	3.21	3.90	0.19	0.24					
	2019_Beltsville	2.75		0.10	2.45	3.11	3.56	0.19	0.08					
	2019_Clayton	2.75		0.10	2.46	3.10	3.75	0.06	0.00					
	Mean	2.77		0.11	2.42	3.16	3.93	0.11	0.12					
Threonine content	2018_Beltsville	1.69		0.07	1.43	1.97	4.31	0.02	0.32	11.5***	1321.41***	231.09***	1.43***	91.32
	2018_Clayton	1.70		0.06	1.46	1.92	3.49	0.17	0.44					
	2019_Beltsville	1.64		0.05	1.43	1.85	3.27	0.21	0.69					
	2019_Clayton	1.64		0.05	1.49	1.87	3.28	0.13	0.12					
	Mean	1.67		0.06	1.45	1.90	3.59	0.13	0.39					

^***^Significant at *P* < 0.001; ^a^: Standard deviation; ^b^: Coefficient of variation; ^c^: *F* value of genotype; ^d^: *F* value of environment; ^e^: *F* value of replication; ^f^: *F* value of genotype x environment

### The QTLs of protein content, oil content, total protein and oil content and amino acid contents

#### Protein content

**Fig. 5:**
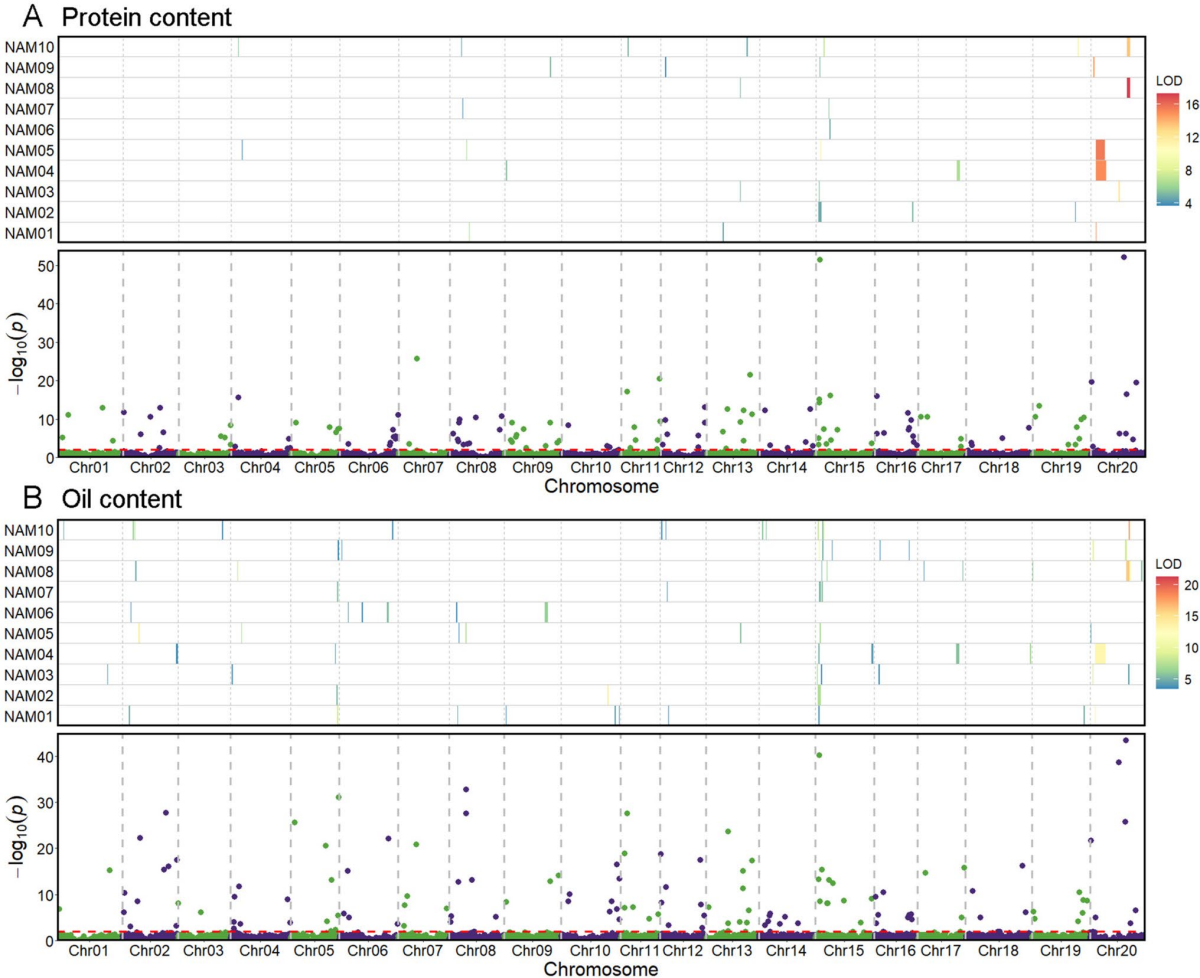
Overview of protein content and oil content QTLs identified by SLM and RTM-GWAS. The rectangles represent the distribution of QTL intervals in soybean genome identified by linkage mapping in a single RIL family. The color scale from blue to red represents the LOD value from low to high. The dots represent the significance level and distribution of QTLs identified by RTM-GWAS based on all NAM families

SLM was performed in each NAM family across four different environments. A total of 99 QTLs associated with protein content were identified in the 10 RIL families, of which 55 QTLs each explained over 10% phenotypic variation. The number of QTLs detected per family ranged from 6 for NAM06 and NAM09 to 15 for NAM04. After integrating overlapping QTLs (marker interval < 1 Mb), a total of 52 nonredundant QTLs were obtained (Table S1), of which 18 QTLs were identified in multiple RIL families or environments. For example, the QTL *qPro-15-1* on chromosome 15 was detected in five RIL families in three environments with an average phenotypic variation explanation (PVE) of 12.36%; the QTL *qPro-20-5* on chromosome 20 was detected in four RIL families in four environments with an average PVE of 22.48%. In addition, the PVE of four QTLs (*qPro-20-2,3,4,5*) on chromosome 20 all exceeded 20%. Based on the RTM-GWAS, a total of 108 QTLs were detected on all 20 chromosomes, with 2 (Chrs 7 and 10) to 9 (Chr. 8) per chromosome. All QTLs related to protein content explained 62.05% of the phenotypic variation. *qPro-20-4 and qPro-15-5* showed extremely significant levels (-Log_10_ *P*: 52.17 and 51.53) and explained 13.49% and 4.17% of the phenotypic variation, respectively. Eleven QTLs (*qPro-2-3*, *qPro-8-4*, *qPro-8-7*, *qPro-12-4*, *qPro-13-2*, *qPro-13-4*, *qPro-13-5*, *qPro-15-5*, *qPro-19-3*, *qPro-20-1* and *qPro-20-4*) each explained more than 1.00% of the phenotypic variation. A total of 58 QTLs, especially the QTLs with high PVE, identified by RTM-GWAS co-located with 35 QTLs identified by SLM (Fig. 5A; Table S2).

#### Oil content

For oil content, a total of 104 QTLs were detected in 10 RIL families by SLM, of which 48 QTLs had PVE over 10%. The number of QTLs detected by each RIL family ranged from 3 for NAM02 to 18 for NAM10. After integrating the overlapping QTLs, a total of 52 unique QTLs were obtained (Table S1), of which 16 were identified in multiple RIL families or environments. The most significant QTL *qOil-20-3* was identified in two RIL families across four environments with an average PVE of 33.75%. *qOil-15-2* and *qOil-15-3* were identified in six RIL families across four environments and explained 15.06% and 25.01% phenotypic variation, respectively. In addition, a total of 128 QTLs related to soybean oil content were identified by RTM-GWAS, which distributed on all 20 chromosomes ranged from 2 (Chrs. 1 and 3) to 11 QTLs (Chr. 15) per chromosome. The PVE of single QTL ranged from 0.01% to 11.71% and they explained 68.93% of phenotypic variation in total. Eleven large-contributing QTLs (*qOil-2-5*, *qOil-5-8*, *qOil-6-4*, *qOil-8-4*, *qOil-8-5*, *qOil-12-5*, *qOil-15-2*, *qOil-15-4*, *qOil-20-1*, *qOil-20-3* and *qOil-20-5*) each explained more than 1.00% of the phenotypic variation. A total of 72 QTLs detected by RTM-GWAS co-located with 43 QTLs detected by SLM, i.e., most QTLs identified by SLM and RTM-GWAS were located in the same or adjacent regions (Fig. 5B; Table S2),

#### Total protein and oil content

SLM identified a total of 34 nonredundant QTL controlling total protein and oil content in 10 RIL families (Table S1). RTM-GWAS identified 119 QTLs, of which 51 were co-located with 28 QTLs from SLM (Fig. S2; Table S2). The most significant QTL *qPro_Oil-8-4* (-Log_10_ *P*: 64.88) detected by RTM-GWAS was co-located by SLM in five RIL families across four environments. Seven co-located QTLs (*qPro_Oil-8-4*, *qPro_Oil-12-2*, *qPro_Oil-12-4*, *qPro_Oil-13-7*, *qPro_Oil-17-3*, *qPro_Oil-19-6*, *qPro_Oil-20-4*) each explained more than 1.00% of the phenotypic variation in RTM-GWAS analysis.

#### Amino acid contents

A total of 34, 35, 43 and 78 QTLs identified by RTM-GWAS co-localized with 22, 20, 28 and 44 QTLs identified by SLM, and these QTLs were significantly associated with methionine, cysteine, lysine and threonine content, respectively (Fig. S3; Table S2). Some QTLs had large effects, such as *qMet-8-1*, *qMet-15-2*, *qMet-19-6* and *qMet-20*-2 for methionine content; *qCys-8-2*, *qCys-13-4*, *qCys-15-2*, *qCys-19-1* and *qCys-20-1* for cysteine content; *qLys-8-4*, *qLys-13-2*, *qLys-13-4*, *qLys-13-5*, *qLys-15-1*, *qLys-19-3* and *qLys-20-2* for lysine content; *qThr-13-2*, *qThr-13-6*, *qThr-13-8*, *qThr-15-2*, *qThr-19-4*, *qThr-20-1* and *qThr-20-3* for threonine content. Interestingly, the most significant SNP locus *chr20_28741905* associated with protein content was also associated with all the four amino acid contents.

### QTL-allele effect matrix of protein, oil and amino acid contents

#### Protein content

**Fig. 6:**
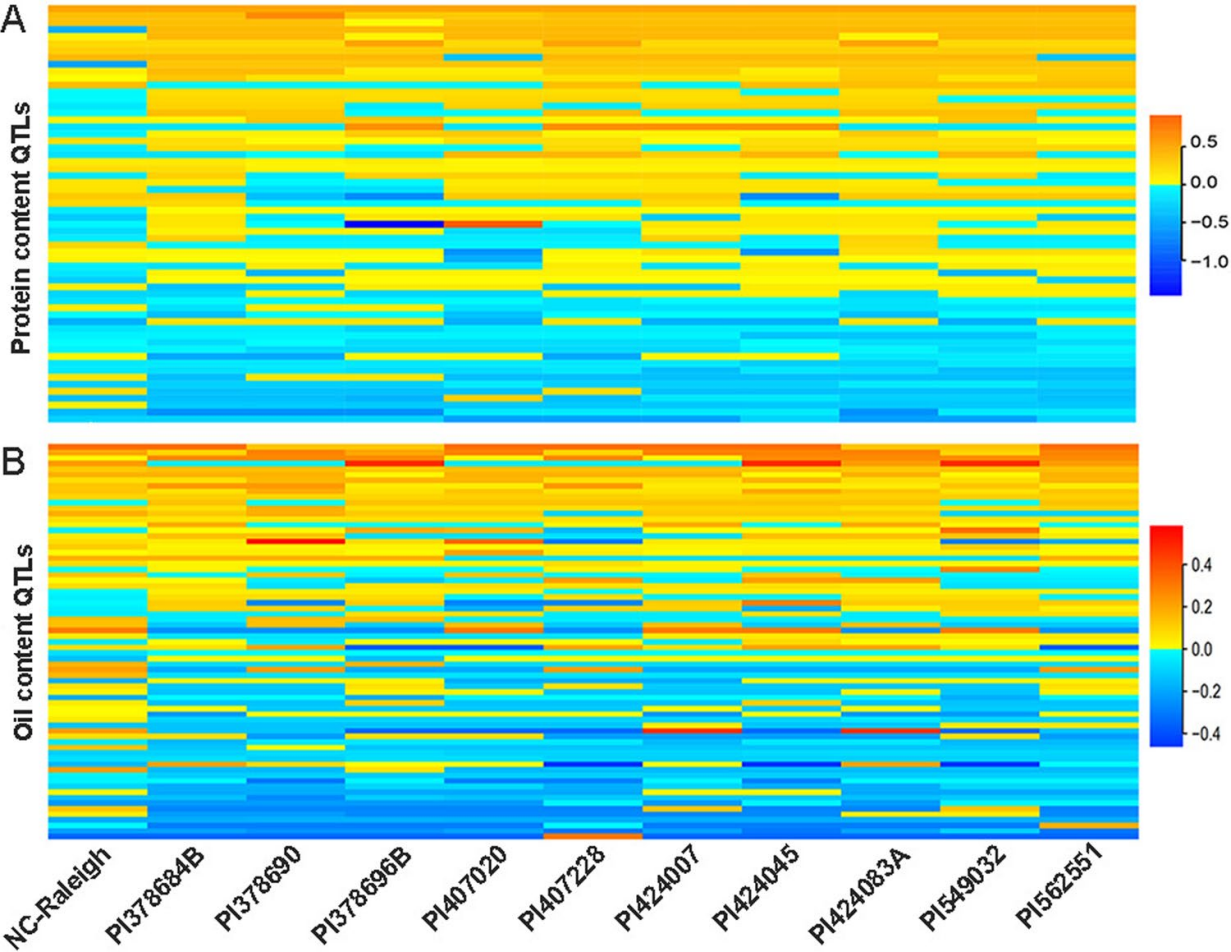
The allelic effect matrix of QTLs for protein content and oil content of 11 parents in the NAM population. Red and blue represent positive and negative allelic effect value, respectively

RTM-GWAS identified 58 protein QTLs located at the same position as the QTL identified by SLM, including 18 QTLs associated with multiple SNPs and 40 QTLs with a single SNP. There were 142 haplotype alleles from the 58 QTLs and the allelic effects were between − 1.46 and 0.91 (Table S3). The protein content QTL-allele effect matrix of 11 parents showed wild soybean parents contained more positive effect alleles than the *G. max* common parent (Fig. 6A; Table S4). A total of 21 QTLs showed positive allelic effects in one or more wild parents, of which four QTLs (*qPro-20-4*, *qPro-15-5*, *qPro-19-3* and *qPro-13-5*) including one newly detected, *qPro-19-3*, showed significant contribution (PVE > 1.00%) based on RTM-GWAS (Table S5). The result indicated that wild soybean had potential to improve elite soybean protein content.

#### Oil content

The 72 oil content QTLs detected by RTM-GWAS and SLM, including 24 QTLs associated with multiple SNPs and 48 QTLs with a single SNP. The 72 QTLs contained 181 haplotype alleles and the allelic effects ranged from − 0.46 to 0.58 (Table S3). A QTL allelic effect matrix for oil content was constructed for 11 parents. Unlike the protein content, the common cultivated soybean parent contained more positive alleles than the wild soybean parents (Fig. 6B; Table S4). Nevertheless, 15 QTLs showed positive effects in wild parents, including six newly detected QTLs (Table S5). Five QTLs associated with both protein content and oil content showed opposite allelic effects in the 11 parents, only one QTL (*Chr20_24615630*) showed positive allelic effects for both protein content and oil content in some wild soybean parents.

#### Total protein and oil content

For total protein and oil content, 51 QTLs detected by RTM-GWAS and SLM, including 11 QTLs associated with multiple SNPs and 40 QTLs with a single SNP. The 51 QTLs contained 117 haplotype alleles and the allelic effects ranged from − 0.81 to 0.56 (Table S3). A total of 19 QTLs showed positive allelic effects only from wild parents (ranged from 0.04 to 0.56) including two large-contributing QTLs (*qPro_Oil-20-4* and *qPro_Oil-19-6*) (Fig. S4; Table (S4, S5). Three QTLs (*Chr11_5074720*, *Chr20_1658893*, *Chr20_28741905*) related to protein content and total protein and oil content showed consistent negative or positive allelic effect trend across 11 parents. However, no major co-located QTL between oil content and total protein and oil content was identified.

#### Amino acid contents

The 34 methionine content QTLs detected by RTM-GWAS and SLM, including 7 QTLs related to multiple SNPs and 27 QTLs to a single SNP, the effect range of 83 haplotype alleles was − 0.0153 to 0.0125 (Table S3). A total of 11 QTLs showed positive allelic effects only from wild parents (ranged from 0.0005 to 0.0051) including three large-contributing QTLs (*qMet-8-1*, *qMet-20-2* and *qMet-15-2*) (Fig. S4; Table S4, S5). The 35 cysteine content QTLs detected by RTM-GWAS and SLM, including 85 haplotype alleles from eight multiple-SNP QTLs and 27 single-SNP QTLs, and the allelic effects ranged from -0.0111 to 0.0140 (Table S3). A total of 16 QTLs showed positive allelic effects from wild parents (ranged from 0.0007 to 0.0110), of which three (*qCys-20-1*, *qCys-13-4* and *qCys-15-2*) showed large contribution (Fig. S4; Table S4, S5). The 43 lysine content QTLs detected by RTM-GWAS and SLM, including 13 QTLs associated with multiple SNPs and 30 QTLs with a single SNP. The 43 QTLs contained 107 haplotypic alleles and the allelic effects ranged from − 0.0650 to 0.0354 (Table S3). Among them, 18 QTLs showed positive allelic effects from wild parents (ranged from 0.0009 to 0.0289), including 5 large-contributing QTLs (*qLys-20-2*, *qLys-15-1*, *qLys-13-4*, *qLys-13-5* and *qLys-19-3*) (Fig. S4; Table S4, S5). For threonine content, 78 QTLs detected by RTM-GWAS and SLM, containing 181 haplotype alleles from 17 QTLs associated with multiple SNPs and 61 QTLs with a single SNP, with allelic effects ranged from − 0.0209 to 0.0192 (Table S3). In total, 24 QTLs showed positive allelic effects from wild parents (ranged from 0.0002 to 0.0160) including four large-contributing QTLs (*qThr-20-3*, *qThr-15-2*, *qThr-13-6* and *qThr-13-8*) (Fig. S4; Table S4, S5). The SNPLDB locus *BLK_Chr13_ 37312231_37400090* was associated with all four amino acids and showed negative allelic effects from most wild parents, the SNP at *Chr20_28741905*, which was also associated with all four amino acids, showed negative effects from common parent but positive effects from wild parents, and the SNP at *Chr11_5074720*, which was associated with methionine, lysine and threonine, showed positive effects from 9 of the 11 parents. These three QTLs were also associated with protein content, and their allelic effect was positively associated with the effects on protein content and amino acid content from the parents.

From the allelic effect matrix of all trait QTLs in 1107 RILs and parents (Fig. 6, S4, S5; Table S4, S6), no NAM line/parent had a completely negative or completely positive allelic effect on traits from QTL regardless of its content. Therefore, the improvement of protein content, oil content and amino acid content had huge potential, and the utilization of superior or complimentary variation from low and middle content germplasm should not be ignored in breeding.

### Candidate gene inferred of protein, oil and amino acid contents

#### Protein content

A total of 53 candidate genes were inferred from the genomic regions of the 58 protein content QTLs identified by SLM and RTM-GWAS (Table S7). These candidate genes could be divided into four categories of biological processes. The first category was related to amino acid synthesis and protein metabolism, including cysteine, leucine and arginine biosynthetic process, protein autophosphorylation, protein polymerization, and protein serine and threonine kinase activity. The second category was related to signal transduction and transport process, including signal transduction, transmembrane transport, amino acid transport and protein transport. The third category was related to metabolic pathway, including transcriptional regulation and glucuronoxylan metabolic process. The fourth category was related to ATP binding.

#### Oil content

In addition, a total of 63 oil content candidate genes were inferred (Table S7). These candidate genes were involved in four biological processes. The first category was candidate genes related to lipid synthesis, including lipid storage, lipid biosynthetic process, fatty acid biosynthetic process and acetyl-CoA biosynthetic process. The second category was related to metabolic pathway, including lipid metabolic, glycolysis, carbohydrate metabolic process and oxidation–reduction process. The third category was related to signal transduction and transport process, including intracellular signal transduction, Golgi vesicle transport and lipid transport. The fourth category was indirectly related to oil content, such as embryo development, regulation of meristem growth, photosynthesis and regulation of organ morphogenesis.

#### Amino acid contents

The potential candidate genes for the four amino acids were predicted based on the position of the co-located QTLs (Table S7). A total of 30 candidate genes were predicted to be associated with soybean methionine content, including three genes involved in methionine biosynthetic process. Thirty-one candidate genes were predicted to be associated with soybean cysteine content, of which six genes involved in cysteine biosynthetic process and one gene involved in cysteine-type endopeptidase activity. For soybean lysine content, 37 candidate genes were inferred, including one gene related to lysine biosynthetic process and two genes involved in histone lysine methylation. Additionally, 70 candidate genes were predicted to be associated with soybean threonine content, including 12 genes related to protein serine or threonine kinase/phosphatase activity and one gene related to threonine-type endopeptidase activity.

## Discussion

Wild soybean was not frequently used in breeding programs due to the undesirable traits, such as shattering pods, vining growth habits, and small seed size. Although we can simply backcross newly identified alleles into *G. max* to improve these traits, backcrossing wild soybean alleles can be difficult in practice. Taliercio et al. (2023) reported that choice of wild parents and selection for larger seed in early generations of large populations can do better than backcrossing to speed up the breeding process and maximize recovery of the wild genome. The most recent germplasm releases show that not all protein advances from wild soybean result in yield decreases (Eickholt et al. 2019; Fallen et al. 2024). This result further emphasizes that newly identified alleles and candidate genes may have benefit in the “real world.”

Although many QTL controlling soybean seed composition traits have been mapped, most QTL have been identified in US elite germplasm, omitting a vast pool of potentially favorable alleles for these traits from wild soybean. It is important to mine this gene pool. In addition, fine mapping seed composition trait QTL from wild soybean will help geneticists and breeders to identify these genes and to introgress desired genes using tightly linked markers.

Soybean QTL mapping is usually based on GWAS of natural populations or linkage mapping in segregating populations. NAM makes full use of historical REs of the parents and newly generated REs in segregating families by combining the strengths of both linkage mapping and association mapping (Gage et al. 2020). In NAM populations, the ideal set of parents shall maximize genetic diversity to ensure that the populations exhibit large trait variation (Gage et al. 2020). In this study, the 10 wild soybean accessions have different protein, oil and amino acid contents and come from different countries and different maturity groups, while NC-Raleigh has excellent yield potential, high oil content and wide environmental adaptability in the southern USA. The high level of genetic and phenotypic diversity of all traits in the soybean NAM population provides a good prerequisite for further genetic analysis of the traits (Table 5; Fig. 3).

### Genetic characterization of the soybean NAM population

RE is a key factor that determines the resolution of QTL mapping and is also a major factor that weakens intrachromosomal LD (Flint-Garcia et al. 2003; Anderson et al. 2018; Zou et al. 2024). By creating cultivated soybean and wild soybean-derived populations, it provides an opportunity to detect the recombination events, thus increasing effective recombination and reducing LD. In this NAM population, the average number of REs was approximately 110 per RIL, 78.9% (95,010) of the REs occurred in at least two RILs in a family, and only 22% (25,467) of the REs (~ 24 REs per line) were unique to one RIL in each family. In the NAM population formed by crossing 40 diverse cultivated soybean accessions, the average number of REs per RIL was 58 (Song et al. 2017), which was less than the REs observed in this study, indicating that the NAM population constructed from wild soybean had increased REs that should lead to higher-resolution QTL mapping. Previous studies showed that the average number of REs per line in maize and rapeseed NAM populations was only 29 and 41, respectively (Kump et al. 2011; Hu et al. 2018).

In this study, only 7.16% (1892) of the SNPs exhibited SD but there was no significant difference in SD between euchromatic and heterochromatic regions, which was consistent with the study of Song et al. (2017) on *G. max*. Approximately 80% of the SNPs favored wild parent alleles, and only 20% of the SNPs favored the common cultivated soybean parent. Other studies showed 70.69% and 58.00% SNPs with SD favoring common parent in NAM population for rapeseed (Hu et al. 2018) and soybean (Song et al. 2017), respectively. We speculate that some alleles from wild parent showed advantages over alleles from common parent of cultivated soybean. This may be related to stress resistance, hybrid vigor or reproductive barriers. The wild soybeans have developed resistance to abiotic and biotic stresses that are necessary to survive in the wild for hundreds of years. Most of those resistances are generally lost through the domestication process. Hybrid plants produced by crossing between genetically diverse parents often exhibit increased vigor and performance compared to their parents (Taliercio et al. 2017). The increased genetic diversity from the wild parent can help enhance the performance of offspring, resulting in healthier and more robust plants. In some cases, reproductive barriers between cultivated and wild soybeans can lead to differences in allele transmission, there may be mechanisms that enhance retention of alleles from one parent over the other. These mechanisms may include preferential chromosome pairing or gamete selection during fertilization (Kianian and Quiors 1992; Matsubara et al. 2011).

As the residual heterozygous lines in the NAM population can be exploited for subsequent QTL fine mapping, we also calculated the percentage of heterozygous SNP loci in the F_6_ RILs of the NAM population, and the ratio of 2.42% was close to the theoretical value 3.13% for F_6_ plants (Table 4). No significant RH difference was found between the euchromatic and heterochromatic regions. In maize NAM population, higher levels of heterozygosity were observed near centromeres (McMullen et al. 2009). Differences in the way crops are propagated may be a factor in this difference.

### Association of traits and allelic effects

Different degrees of correlation were identified among all traits. The significant correlations between these traits corresponded with allelic effects of co-located QTLs between the traits. For example, for six co-located QTLs controlling protein content and oil content, 77.27% showed opposite allelic effects between the two traits in the 11 parents. For 15 co-located QTLs associated with protein content and lysine content and 18 QTLs associated with protein content and threonine content, 91.52% and 87.88% showed consistent allelic effect trends between the two traits, respectively. However, for five co-located QTLs related to oil content and lysine content and seven QTLs related to oil content and threonine content, 80.00% and 74.03% showed opposite allelic effects between the two paired traits in the 11 parents, respectively.

### QTLs for all traits in comparison with those reported in the literature

#### Protein and oil contents

Numerous QTLs for protein and oil content have been mapped using both biparental populations and germplasm soybean populations (Van and McHale 2017). In this study, we performed linkage mapping analysis of traits in each of the ten RIL families and RTM-GWAS for traits across the entire NAM population. Through SLM analysis, 99 protein content QTLs and 104 oil content QTLs were identified in 10 RIL families, and both integrated into 52 nonredundant QTLs (Table S1). Many of these QTLs were detected in multiple RIL families or environments. At these loci, the wild parents contained many alleles that had positive effects on protein content but negative effects on oil content. Meanwhile, RTM-GWAS detected 108 protein content QTLs and 128 oil content QTLs on all 20 chromosomes, which explained 62.05% and 68.93% of the phenotypic variation, respectively. The results obtained by the two methods were in good agreement, 53.7% (58/108) of the protein content QTLs and 56.3% (72/128) of the oil content QTLs detected by RTM-GWAS fell within the confidence interval of the QTL detected by SLM mapping. Many previous studies mapped major QTLs related to soybean protein and oil contents on chromosomes 15 and 20 (Diers et al. 1992; Lu et al. 2013; Warrington et al. 2015; Pandurangan et al. 2012; Kim et al. 2016; Hwang et al. 2014; Lee et al. 2019). The top-ranked significant QTLs in our study were also mapped to these known regions, for example, the most significant QTL for protein content *qPro-20-4* on chromosome 20 as well as *qPro-15-5* on chromosome 15. Recently, the genes controlling protein content at *qPro-20-4* on chromosome 20 (Goettel et al. 2022; Fliege et al. 2022) and at *qPro-15-5* on chromosome 15 (Zhang et al. 2020) were cloned. The most significant QTL for seed oil content (*qOil-20-5*) was also for protein content (*qPro-20-6*), but the estimated effect of the wild parent allele was positive for protein content but negative for oil content, like previous reports (Bandillo et al. 2015; Lee et al. 2019). Currently, more than 240 QTLs for protein content and oil content have been reported per SoyBase (http://www.soybase.org), comparing the genomic positions of these QTLs to the QTLs identified in this study, among the 58 protein content QTLs co-localized by SLM and RTM-GWAS, 40 QTLs were located at or close to QTLs documented in SoyBase (Table S2), but 18 were new. The newly detected QTLs that contributed significantly to protein content included *qPro-19-3* (2.00%), *qPro-12-2* (0.57%) and *qPro-20-6* (0.48%). Among the 72 oil content QTLs co-located by SLM and RTM-GWAS, 51 were previously mapped QTLs, 21 were newly detected (Table S2), including QTLs *qOil-2-5* (1.44%), *qOil-6-4* (2.09%) *and qOil-15-2* (3.20%), which contributed significantly.

The wild parents contained more alleles with positive effects on protein content and negative effects on oil content (Fig. 6; Table S4, S5). In the QTL-allele effect matrix, all the parents and RILs contained positive and negative effect alleles for protein content and oil content. The allelic effect among the parents ranged from − 1.46 to 0.91 for protein content and -0.46 to 0.58 for oil content, indicating that each parent has the potential to improve seed composition. We observed 21 protein content and 15 oil content positive alleles in wild soybean that can be exploited for genetic improvement of cultivated soybean. Although there is a significant negative correlation between protein and oil content, 19 QTLs related to total protein and oil content exhibited positive allelic effects from wild parents, which can be used to reduce the negative correlation between protein and oil contents and improve the overall composition traits of soybean.

#### Total protein and oil content

Since approximately 60% of soybean value comes from soybean meal and the remainder from oil (Pettersson and Pontoppidan 2013), the total protein and oil content in soybean seed appears to be more important than just the protein content or oil content separately. In addition, since protein content is negatively correlated with oil content, identifying and utilizing QTL that can increase total protein and oil content may be an approach to reduce the impact of a negative correlation between protein content and oil content. In this study, 43.7% (52/119) QTLs for the total protein and oil content detected by RTM-GWAS fell within the confidence interval of the QTL detected by SLM mapping. Seven newly detected QTLs contributed significantly, including *qPro_Oil-20-4* (7.24%), *qPro_Oil-8-4* (5.55%), *qPro_Oil-13-7* (3.15%), *qPro_Oil-12-4* (2.27%), *qPro_Oil-17-3* (1.70%), *qPro_Oil-19-6* (1.44%) and *qPro_Oil-12-2* (1.16%). Two top-ranked significant QTLs, *qPro_Oil-8-4* and *qPro_Oil-20-4*, overlapped with QTLs controlling protein content but not oil content. There have been few genetic studies on total protein and oil content (Chen et al. 2007), and this study provides new QTLs related to this trait for further research.

#### Amino acid contents

We identified several novel QTLs associated with the four amino acids (Table S2). Approximately 40.0% (34/85) of the methionine QTLs, 41.2% (35/85) of the cysteine QTLs, 39.4% (43/109) of the lysine QTLs and 59.5% (78/131) of the threonine QTLs detected by RTM-GWAS fell within the confidence interval of the SLM mapping (Table S2). Interestingly, the most significant QTLs detected for methionine, lysine and threonine were all at the same genomic locus on chromosome 20 (*Chr20_28741905*), which was also the QTL for protein content. The correlated mapping results for these three amino acids may be due to the fact that methionine, lysine and threonine were part of the aspartate family of amino acids synthesized from the same precursor (Warrington et al. 2015). Several studies have reported the genomic regions associated with amino acid content in soybean. For example, Warrington et al. (2015) identified four QTLs associated with methionine (Chrs 6, 09, 10 and 20), one QTL for cysteine (Chr 10), two QTLs for lysine (Chrs 9 and 20) and four QTLs for threonine (Chrs 1, 9, 17 and 20) based on a biparental RIL population. Lee et al. (2019) identified eight QTLs associated with amino acids methionine, cysteine, lysine and threonine on eight chromosomes, although these QTLs were not stable across environments. In this study, methionine QTLs *qMet-4-2*, *qMet-5-1*, *qMet-7-1*, *qMet-15-4*, *qMet-15-5*, *qMet-20-2* were detected in similar genomic regions of previous reports (Panthee et al. 2006a, b; Wang et al. 2015; Warrington et al. 2015; Kastoori Ramamurthy et al. 2014). The lysine QTLs *qLys-15-5* and *qLys-20-3* were in two QTL regions reported before (Panthee et al. 2006a; Lee et al. 2019). Twelve QTLs associated with threonine were mapped to similar genomic regions in previous studies (Panthee et al. 2006a; Warrington et al. 2015; Lee et al. 2019). Some newly detected QTLs contributed significantly, such as *qMet-8-1*(24.89%), *qMet-19-6* (4.08%) and *qMet-15-2* (1.09%) for methionine content; *qCys-8-2* (17.92%), *qCys-20-1* (7.54%), *qCys-19-1* (3.49%), *qCys-13-4* (1.52%) and *qCys-15-2* (1.29%) for cysteine content; *qLys-20-2* (25.38%), *qLys-8-4* (13.59%), *qLys-15-1* (3.80%), *qLys-13-2* (2.57%), *qLys-13-4* (1.54%), *qLys-13-5* (1.54%) and *qLys-19-3* (1.25%) for lysine content; *qThr-15-2* (3.90%), *qThr-19-4* (2.04%), *qThr-13-2* (1.99%), *qThr-13-6* (1.90%), *qThr-20-1* (1.23%) and *qThr-13-8* (1.07%) for threonine content.

### Candidate gene analysis of QTLs for all traits identified by SLM and RTM-GWAS

#### Protein content

The flanking genes surrounded by co-located QTLs were considered as potential candidate genes. We only focused on the genes whose functional annotation is related to the studied traits. A total of 53 candidate genes associated with soybean protein content were detected (Table S7). The genes at the most significant QTL for protein content *qPro-20-4* as well as *qPro-15-5* have been cloned. *POWR1* (*Glyma.20G85100*) is a CCT-domain gene that can significantly affect soybean protein and oil content by regulating seed nutrient transport and lipid metabolism (Goettel et al. 2022). *GmSWEET39* (*Glyma.15G049200*) is a seed coat-preferentially expressed sugar transporter gene, which may regulate the accumulation of oil and protein by affecting the sugar transport from maternal seed coat to the filial embryo (Zhang et al. 2020). Some of these candidate genes were specifically expressed in seeds (Fig S6; Table S8), such as *Glyma.02G145700* (*qPro-2-2*) and *Glyma.19G164900* (*qPro-19-5*) (associated with nutrient reservoir activity). *Glyma.02G145700* is homologous with Arabidopsis *PAP85* gene, which encodes a vicilin-like seed storage protein and expressed at the stage of seed development (Parcy et al. 1994). *Glyma.19G164900* encodes a glycinin (11S) seed storage protein, it is an ortholog of the Arabidopsis *CRA1* whose subunits are assembled and deposited in protein storage vacuoles (Wan et al. 2007).

#### Oil content

In addition, a total of 63 candidate genes associated with soybean oil content were inferred (Table S7). The candidate gene of large-contributing QTL *qOil-8-4*, *Glyma.08G183500*, has been identified as an ortholog of the Arabidopsis *SWEET15* gene. *AtSWEET15* mutants display serious seed defects, including embryo development retardation, seed weight reduction, starch and lipid content reduction, resulting in seed shrinkage (Chen et al. 2015). *Glyma.10G255100* (*qOil-10-6*) is an ortholog of the Arabidopsis *PKP1* gene, which is important for seed oil biosynthesis with significantly increased expression in maturing seeds. *AtPKP1* mutants were unable to accumulate storage oil to the same extent as the wild type (Andre and Benning 2007). *Glyma.15G046300* (large-contributing QTL *qOil-8-4*) and *Glyma.17G251000* (*qOil-17-3*) are homologous with Arabidopsis *KCS7* and *KCS4*, respectively. The condensing enzyme β-Keto-acyl-CoA Synthase (KCS) plays an important role in biosynthesis of very-long-chain fatty acids (VLCFAs), which can be incorporated in seeds triacylglycerols toward the accumulation of storage lipids (Batsale et al. 2021). The candidate genes *Glyma.10G203400* (*qOil-10-4*) and *Glyma.17G086400* (*qOil-17-1*) related to lipid metabolic and storage were specifically expressed in seeds (Fig S6; Table S8).

#### Amino acid contents

For the four amino acids, 30, 31, 37, 70 candidate genes were predicted to be associated with soybean methionine, cysteine, lysine and threonine content, respectively (Table S7). For soybean methionine content, *Glyma.08G111700* was predicted as candidate gene of the largest-contributing QTL *qMet-8-1* (24.89%) and annotated to function in methionine biosynthesis process. Its homologous gene *SUMO2* in Arabidopsis can covalently be attached to various intracellular protein targets, much like ubiquitination, leading to posttranslational modification of those targets (Saracco et al. 2007). *Glyma.20G129700* (*qMet-20-3*) is an ortholog of the Arabidopsis *MTK* gene, which encodes 5-methylthioribose kinase and involved in methionine cycle (Bürstenbinder et al. 2007). For soybean cysteine content, *Glyma.19G119200* was predicted as candidate gene of the most significant QTL *qCys-19-1* and annotated to be involved in cysteine biosynthesis. *Glyma.19G119200* is an ortholog of the Arabidopsis *CS26* gene, which performs catalytic and regulatory roles in the cysteine biosynthesis pathway (Singh et al. 2021). For soybean lysine content, *Glyma.06G053000* (*qLys-6-1*) was annotated as involved in lysine biosynthetic process via diaminopimelate. For soybean threonine content, *Glyma.03G037700* (*qThr-3-1*) was annotated as involved in cellular amino acid metabolic process, which is an ortholog of the Arabidopsis *ASP1* gene. *ASP1* mediates aspartic acid biosynthesis, which is the precursor of threonine synthesis in organisms (De La Torre et al. 2014). Some candidate genes (such *Glyma.08G111700*, *Glyma.08G109800*, *Glyma.11G067900, Glyma.12G221100*) were specifically expressed in seeds, indicating that they may play an important role in seed amino acid accumulation (Fig S6; Table S8).

## Conclusions

A NAM population consisting of 10 RIL families was developed by crossing 10 wild soybeans with a cultivated soybean. Higher number of recombinant events than population derived from cultivated soybeans only were observed. Segregation distortion in nearly all families significantly favored the alleles from the wild soybean parents. RIL residual heterozygosity covering the entire genome provided important genetic resources for fine mapping of QTL in subsequent populations. We also determined the scope of effects of the QTLs controlling the contents of seed protein, oil and sulfur-containing amino acids (cysteine, methionine), lysine and threonine in wild soybean, detected novel loci showing large positive effects on the seed composition traits from wild soybean and candidate genes controlling the traits. This is the first study to reveal genetic characteristics of a wild soybean-derived population and the QTL landscape and extent of effects from a diverse set of wild soybean parents and candidate genes controlling the traits. Information from this study provides new knowledge about wild soybean traits and will promote the use of wild soybeans to improve seed composition traits of cultivated soybeans.

## Supplementary Information

Below is the link to the electronic supplementary material.

Supplementary file1 (TIF 6536 KB)

Supplementary file2 (TIF 1045 KB)

Supplementary file3 (TIF 1763 KB)

Supplementary file4 (TIF 9500 KB)

Supplementary file5 (TIF 6544 KB)

Supplementary file6 (XLSX 42 KB)

Supplementary file7 (XLSX 104 KB)

Supplementary file8 (XLSX 55 KB)

Supplementary file9 (XLSX 55 KB)

Supplementary file10 (XLSX 36 KB)

Supplementary file11 (XLSX 1475 KB)

Supplementary file12 (XLSX 58 KB)

Supplementary file13 (XLSX 42 KB)

## References

[CR1] Anderson SL 2nd, Mahan AL, Murray SC, Klein PE (2018) Four parent maize (FPM) population: effects of mating designs on linkage disequilibrium and mapping quantitative traits. Plant Genome 11(2):170102 10.3835/plantgenome2017.11.0102 PMC12810144 30025026

[CR2] Andre C, Benning C (2007) Arabidopsis seedlings deficient in a plastidic pyruvate kinase are unable to utilize seed storage compounds for germination and establishment. Plant Physiol 145(4):1670–1680 17965177 10.1104/pp.107.108340 PMC2151681

[CR3] Bandillo N, Jarquin D, Song Q, Nelson R, Cregan P, Specht J, Lorenz A (2015) A population structure and genome-wide association analysis on the USDA soybean germplasm collection. Plant Genome. 10.3835/plantgenome2015.04.0024 33228276 10.3835/plantgenome2015.04.0024

[CR4] Batsale M, Bahammou D, Fouillen L, Mongrand S, Joubès J, Domergue F (2021) Biosynthesis and functions of very-long-chain fatty acids in the responses of plants to abiotic and biotic stresses. Cells 10(6):1284 34064239 10.3390/cells10061284 PMC8224384

[CR5] Bouchet S, Olatoye MO, Marla SR, Perumal R, Tesso T, Yu J, Tuinstra M, Morris GP (2017) Increased power to dissect adaptive traits in global sorghum diversity using a nested association mapping population. Genetics 206(2):573–585 28592497 10.1534/genetics.116.198499 PMC5499173

[CR6] Brim CA (1966) A modified pedigree method of selection in soybeans1. Crop Science 6(2):220

[CR7] Brummer EC, Graef GL, Orf J, Wilcox JR, Shoemaker RC (1997) Mapping QTL for seed protein and oil content in eight soybean populations. Crop Sci 37(2):370–378

[CR8] Bürstenbinder K, Rzewuski G, Wirtz M, Hell R, Sauter M (2007) The role of methionine recycling for ethylene synthesis in Arabidopsis. Plant J 49(2):238–249 17144895 10.1111/j.1365-313X.2006.02942.x

[CR9] Burton JW, Carter TE, Fountain MO, Bowman DT (2006) Registration of “NC-Raleigh” soybean. Crop Sci 46(6):2710–2711

[CR10] Chen Q, Zhang Z, Liu C, Xin D, Qiu H, Shan D, Shan C, Hu G (2007) QTL analysis of major agronomic traits in soybean. Agric Sci China 6(4):399–405

[CR11] Chen L, Lin I, Qu X, Sosso D, McFarlane HE, Londoño A, Samuels AL, Frommer WB (2015) A cascade of sequentially expressed sucrose transporters in the seed coat and endosperm provides nutrition for the Arabidopsis embryo. Plant Cell 27(3):607–619 25794936 10.1105/tpc.114.134585 PMC4558658

[CR12] Chen L, Yang S, Araya S, Quigley C, Taliercio E, Mian R, Specht JE, Diers BW, Song Q (2022) Genotype imputation for soybean nested association mapping population to improve precision of QTL detection. Theor Appl Genet 135(5):1797–1810 35275252 10.1007/s00122-022-04070-7 PMC9110473

[CR13] Chung J, Babka HL, Graef GL, Staswick PE, Lee DJ, Cregan PB, Shoemaker RC, Specht JE (2003) The seed protein, oil, and yield QTL on soybean linkage group I. Crop Sci 43(3):1053–1067

[CR14] De La Torre F, Cañas RA, Pascual MB, Avila C, Cánovas FM (2014) Plastidic aspartate aminotransferases and the biosynthesis of essential amino acids in plants. J Exp Bot 65(19):5527–5534 24902885 10.1093/jxb/eru240

[CR15] Diers BW, Keim P, Fehr WR, Shoemaker RC (1992) RFLP analysis of soybean seed protein and oil content. Theor Appl Genet 83:608–612 24202678 10.1007/BF00226905

[CR16] Diers BW, Specht J, Rainey KM, Cregan P, Song Q, Ramasubramanian V, Graef G, Nelson R, Schapaugh W, Wang D, Shannon G, McHale L, Kantartzi SK, Xavier A, Mian R, Stupar RM, Michno JM, An YC, Goettel W, Ward R, Fox C, Lipka AE, Hyten D, Cary T, Beavis WD (2018) Genetic architecture of soybean yield and agronomic traits. G3 Genes Genomes Genet 8(10):3367–3375 10.1534/g3.118.200332 PMC6169381 30131329

[CR17] Diers BW, Specht JE, Graef GL, Song Q, Rainey KM, Ramasubramanian V, Liu X, Myers CL, Stupar RM, An YQ, Beavis WD (2023) Genetic architecture of protein and oil content in soybean seed and meal. The Plant Genome 16(1):e20308 36744727 10.1002/tpg2.20308 PMC12807260

[CR18] Eickholt D, Carter TE, Taliercio E, Dickey D, Dean LO, Delheimer J, Li Z (2019) Registration of USDA-*Max*×*Soja* Core Set-1: Recovering 99% of wild soybean genome from PI 366122 in 17 agronomic interspecific germplasm lines. J Plant Regist 13(2):217–236

[CR19] Fallen B, Robertson M, Taliercio E, Mian MR, Carter TE (2024) Registration of high-yielding, high-protein soybean germplasm USDA-N7007 derived from wild soybean PI 366122. J Plant Regist 18(3):538–546

[CR20] Fliege CE, Ward RA, Vogel P, Nguyen H, Quach T, Guo M, Viana JP, Dos Santos LB, Specht JE, Clemente TE, Hudson ME (2022) Fine mapping and cloning of the major seed protein quantitative trait loci on soybean chromosome 20. Plant J 110(1):114–128 34978122 10.1111/tpj.15658 PMC9303569

[CR21] Flint-Garcia SA, Thornsberry JM, Buckler ES IV (2003) Structure of linkage disequilibrium in plants. Annu Rev Plant Biol 54(1):357–374 14502995 10.1146/annurev.arplant.54.031902.134907

[CR22] Fragoso CA, Moreno M, Wang Z, Heffelfinger C, Arbelaez LJ, Aguirre JA, Franco N, Romero LE, Labadie K, Zhao H, Dellaporta SL (2017) Genetic architecture of a rice nested association mapping population. G3 Genes Genomes Genet 7(6):1913–1926 10.1534/g3.117.041608 PMC5473768 28450374

[CR23] Gage JL, Monier B, Giri A, Buckler ES (2020) Ten years of the maize nested association mapping population: impact, limitations, and future directions. Plant Cell 32(7):2083–2093 32398275 10.1105/tpc.19.00951 PMC7346555

[CR24] George AA, De Lumen BO (1991) A novel methionine-rich protein in soybean seed: identification, amino acid composition, and n-terminal sequence. J Agric Food Chem 39(1):224–227

[CR25] Goettel W, Zhang H, Li Y, Qiao Z, Jiang H, Hou D, Song Q, Pantalone VR, Song BH, Yu D, An YQ (2022) POWR1 is a domestication gene pleiotropically regulating seed quality and yield in soybean. Nat Commun 13(1):3051 35650185 10.1038/s41467-022-30314-7 PMC9160092

[CR26] He J, Meng S, Zhao T, Xing G, Yang S, Li Y, Guan R, Lu J, Wang Y, Xia Q, Yang B (2017) An innovative procedure of genome-wide association analysis fits studies on germplasm population and plant breeding. Theor Appl Genet 130:2327–2343 28828506 10.1007/s00122-017-2962-9

[CR27] Hu J, Guo C, Wang B, Ye J, Liu M, Wu Z, Xiao Y, Zhang Q, Li H, King GJ, Liu K (2018) Genetic properties of a nested association mapping population constructed with semi-winter and spring oilseed rapes. Front Plant Sci 9:1740 30534135 10.3389/fpls.2018.01740 PMC6275288

[CR28] Hwang EY, Song Q, Jia G, Specht JE, Hyten DL, Costa J, Cregan PB (2014) A genome-wide association study of seed protein and oil content in soybean. BMC Genomics 15:1–12 24382143 10.1186/1471-2164-15-1 PMC3890527

[CR29] Hymowitz T (1970) On the domestication of the soybean. Econ Bot 24(4):408–421

[CR30] Hymowitz T, Newell CA (1981) Taxonomy of the genusGlycine, domestication and uses of soybeans. Econ Bot 35(3):272–288

[CR31] Hyten DL, Song Q, Zhu Y, Choi IY, Nelson RL, Costa JM, Specht JE, Shoemaker RC, Cregan PB (2006) Impacts of genetic bottlenecks on soybean genome diversity. Proc Natl Acad Sci 103(45):16666–16671 17068128 10.1073/pnas.0604379103 PMC1624862

[CR32] Kastoori Ramamurthy R, Jedlicka J, Graef GL, Waters BM (2014) Identification of new QTLs for seed mineral, cysteine, and methionine concentrations in soybean [*Glycine max* (L.) Merr.]. Mol Breeding 34:431–445

[CR33] Kerley MS, Allee GL (2003) (2003) Modifications in soybean seed composition to enhance animal feed use and value: moving from a dietary ingredient to a functional dietary component. AgBioForum 6(1&2):14–17

[CR34] Khandaker L, Akond M, Liu S, Kantartzi SK, Meksem K, Bellaloui N, Lightfoot DA, Kassem MA (2015) Mapping of QTL associated with seed amino acids content in “MD96-5722” by “Spencer” RIL population of soybean using SNP markers. Food Nutr Sci 6(11):974

[CR35] Kianian SF, Quiros CF (1992) Generation of a Brassica olera-cea composite RFLP map: linkage arrangements among various populations and evolutionary implications. Theor Appl Genet 84(5–6):544–554 24201339 10.1007/BF00224150

[CR36] Kidane YG, Gesesse CA, Hailemariam BN, Desta EA, Mengistu DK, Fadda C, Pè ME, Dell’Acqua M (2019) A large nested association mapping population for breeding and quantitative trait locus mapping in Ethiopian durum wheat. Plant Biotechnol J 17(7):1380–1393 30575264 10.1111/pbi.13062 PMC6576139

[CR37] Kim M, Schultz S, Nelson RL, Diers BW (2016) Identification and fine mapping of a soybean seed protein QTL from PI 407788A on chromosome 15. Crop Sci 56(1):219–225

[CR38] Kump KL, Bradbury PJ, Wisser RJ, Buckler ES, Belcher AR, Oropeza-Rosas MA, Zwonitzer JC, Kresovich S, McMullen MD, Ware D, Balint-Kurti PJ (2011) Genome-wide association study of quantitative resistance to southern leaf blight in the maize nested association mapping population. Nat Genet 43(2):163–168 21217757 10.1038/ng.747

[CR39] La T, Large E, Taliercio E, Song Q, Gillman JD, Xu D, Nguyen HT, Shannon G, Scaboo A (2019) Characterization of select wild soybean accessions in the USDA germplasm collection for seed composition and agronomic traits. Crop Sci 59(1):233–251

[CR40] Lee S, Van K, Sung M, Nelson R, LaMantia J, McHale LK, Mian MR (2019) Genome-wide association study of seed protein, oil and amino acid contents in soybean from maturity groups I to IV. Theor Appl Genet 132:1639–1659 30806741 10.1007/s00122-019-03304-5 PMC6531425

[CR41] Li Y, Li W, Zhang C, Yang L, Chang R, Gaut BS, Qiu L (2010) Genetic diversity in domesticated soybean (*Glycine max*) and its wild progenitor (*Glycine soja*) for simple sequence repeat and single-nucleotide polymorphism loci. New Phytol 188(1):242–253 20618914 10.1111/j.1469-8137.2010.03344.x

[CR42] Liu Y, Zhang Y, Liu X, Shen Y, Tian D, Yang X, Liu S, Ni L, Zhang Z, Song S, Tian Z (2023) SoyOmics: a deeply integrated database on soybean multi-omics. Mol Plant 16(5):794–797 36950735 10.1016/j.molp.2023.03.011

[CR43] Lu W, Wen Z, Li H, Yuan D, Li J, Zhang H, Huang Z, Cui S, Du W (2013) Identification of the quantitative trait loci (QTL) underlying water soluble protein content in soybean. Theor Appl Genet 126:425–433 23052024 10.1007/s00122-012-1990-8

[CR44] Matsubara K, Ebana K, Mizubayashi T, Itoh S, Ando T, Nonoue Y, Ono N, Shibaya T, Ogiso E, Hori K, Fukuoka S, Yano M (2011) Relationship between transmission ratio distortion and genetic divergence in intraspecific rice crosses. Mol Genet Genomics 286(5–6):307–319 21918817 10.1007/s00438-011-0648-6

[CR45] Maurer A, Draba V, Jiang Y, Schnaithmann F, Sharma R, Schumann E, Kilian B, Reif JC, Pillen K (2015) Modelling the genetic architecture of flowering time control in barley through nested association mapping. BMC Genomics 16(1):1–12 25887319 10.1186/s12864-015-1459-7 PMC4426605

[CR46] McMullen MD, Kresovich S, Villeda HS, Bradbury P, Li H, Sun Q, Flint-Garcia S, Thornsberry J, Acharya C, Bottoms C, Brown P (2009) Genetic properties of the maize nested association mapping population. Science 325(5941):737–740 19661427 10.1126/science.1174320

[CR47] Meng L, Li H, Zhang L, Wang J (2015) QTL IciMapping: Integrated software for genetic linkage map construction and quantitative trait locus mapping in biparental populations. Crop J 3(3):269–283

[CR48] Merk HL, Yarnes SC, Van Deynze A, Tong N, Menda N, Mueller LA, Mutschler MA, Loewen SA, Myers JR, Francis DM (2012) Trait diversity and potential for selection indices based on variation among regionally adapted processing tomato germplasm. J Am Soc Hortic Sci 137(6):427–437

[CR49] Murray MG, Thompson W (1980) Rapid isolation of high molecular weight plant DNA. Nucleic Acids Res 8(19):4321–4326 7433111 10.1093/nar/8.19.4321 PMC324241

[CR50] Nichols DM, Glover KD, Carlson SR, Specht JE, Diers BW (2006) Fine mapping of a seed protein QTL on soybean linkage group I and its correlated effects on agronomic traits. Crop Sci 46(2):834–839

[CR51] Pandurangan S, Pajak A, Molnar SJ, Cober ER, Dhaubhadel S, Hernandez-Sebastia C, Kaiser WM, Nelson RL, Huber SC, Marsolais F (2012) Relationship between asparagine metabolism and protein concentration in soybean seed. J Exp Bot 63(8):3173–3184 22357599 10.1093/jxb/ers039 PMC3350928

[CR52] Panthee DR, Pantalone VR, Saxton AM, West DR, Sams CE (2006a) Genomic regions associated with amino acid composition in soybean. Mol Breed 17:79–89

[CR53] Panthee DR, Pantalone VR, Sams CE, Saxton AM, West DR, Orf JH, Killam AS (2006b) Quantitative trait loci controlling sulfur containing amino acids, methionine and cysteine, in soybean seeds. Theor Appl Genet 112:546–553 16341836 10.1007/s00122-005-0161-6

[CR54] Parcy F, Valon C, Raynal M, Gaubier-Comella P, Delseny M, Giraudat J (1994) Regulation of gene expression programs during Arabidopsis seed development: roles of the *ABI3* locus and of endogenous abscisic acid. Plant Cell 6:1567–1582 7827492 10.1105/tpc.6.11.1567 PMC160544

[CR55] Pettersson D, Pontoppidan K (2013) Soybean meal and the potential for upgrading its feeding value by enzyme supplementation. *Soybean-bio-active compounds*, pp 288–307

[CR56] Saracco SA, Miller MJ, Kurepa J, Vierstra RD (2007) Genetic analysis of SUMOylation in Arabidopsis: conjugation of SUMO1 and SUMO2 to nuclear proteins is essential. Plant Physiol 145(1):119–134 17644626 10.1104/pp.107.102285 PMC1976578

[CR57] Sebolt AM, Shoemaker RC, Diers BW (2000) Analysis of a quantitative trait locus allele from wild soybean that increases seed protein concentration in soybean. Crop Sci 40(5):1438–1444

[CR58] Singh RP, Saini N, Sharma G, Rahisuddin R, Patel M, Kaushik A, Kumaran S (2021) Moonlighting biochemistry of cysteine synthase: a species-specific global regulator. J Mol Biol 433(22):167255 34547327 10.1016/j.jmb.2021.167255

[CR59] Song Q, Hyten DL, Jia G, Quigley C, Fickus E, Nelson RL, Cregan PB (2013) Development and evaluation of SoySNP50K, a high-density genotyping array for soybean. PLoS ONE 8:e54985 23372807 10.1371/journal.pone.0054985 PMC3555945

[CR60] Song Q, Jenkins J, Jia G, Hyten DL, Pantalone V, Jackson SA, Schmutz J, Cregan PB (2016) Construction of high resolution genetic linkage maps to improve the soybean genome sequence assembly Glyma1. 01. BMC Genomics 17:1–11 26739042 10.1186/s12864-015-2344-0 PMC4704267

[CR61] Song Q, Yan L, Quigley C, Jordan BD, Fickus E, Schroeder S, Song BH, An YQ, Hyten D, Nelson R, Rainey K (2017) Genetic characterization of the soybean nested association mapping population. Plant Genome. 10.3835/plantgenome2016.10.0109 28724064 10.3835/plantgenome2016.10.0109

[CR62] Song Q, Yan L, Quigley C, Fickus E, Wei H, Chen L, Dong F, Araya S, Liu J, Hyten D, Pantalone V, Nelson RL (2020) Soybean BARCSoySNP6K: An assay for soybean genetics and breeding research. Plant J 104(3):800–811 32772442 10.1111/tpj.14960 PMC7702105

[CR64] Sun Z, Tian P, Wang J (1990) Study on the uses of aphid-resistant character in wild soybean. I. Aphid-resistance performance of F_2_ generation from crosses between cultivated and wild soybeans. Soybean Genet Newsl 17:43–48

[CR65] Taliercio E, Eickholt D, Rouf R, Carter T (2017) Changes in gene expression between a soybean F_1_ hybrid and its parents are associated with agronomically valuable traits. PLoS ONE 12(5):e0177225 28493991 10.1371/journal.pone.0177225 PMC5426663

[CR66] Taliercio E, Eickholt D, Read QD, Carter T, Waldeck N, Fallen B (2023) Parental choice and seed size impact the uprightness of progeny from interspecific *Glycine* hybridizations. Crop Sci 63(4):2184–2195

[CR67] Tuyen DD, Lal SK, Xu DH (2010) Identification of a major QTL allele from wild soybean (Glycine soja Sieb. & Zucc.) for increasing alkaline salt tolerance in soybean. Theor Appl Genet 121(2):229–236 20204319 10.1007/s00122-010-1304-y

[CR68] Van K, McHale LK (2017) Meta-analyses of QTLs associated with protein and oil contents and compositions in soybean [Glycine max (L.) Merr.] seed. Int J Mol Sci 18(6):1180 28587169 10.3390/ijms18061180 PMC5486003

[CR69] Wan L, Ross AR, Yang J, Hegedus DD, Kermode AR (2007) Phosphorylation of the 12 S globulin cruciferin in wild-type and abi1-1 mutant Arabidopsis thaliana (thale cress) seeds. Biochem J 404(2):247–256 17313365 10.1042/BJ20061569 PMC1868800

[CR70] Wang D, Graef GL, Procopiuk AM, Diers BW (2004) Identification of putative QTL that underlie yield in interspecific soybean backcross populations. Theor Appl Genet 108(3):458–467 14504749 10.1007/s00122-003-1449-z

[CR71] Wang X, Jiang G, Song Q, Cregan PB, Scott RA, Zhang J, Yen Y, Brown M (2015) Quantitative trait locus analysis of seed sulfur-containing amino acids in two recombinant inbred line populations of soybean. Euphytica 201:293–305

[CR72] Warrington CV, Abdel-Haleem H, Hyten DL, Cregan PB, Orf JH, Killam AS, Bajjalieh N, Li Z, Boerma HR (2015) QTL for seed protein and amino acids in the Benning × Danbaekkong soybean population. Theor Appl Genet 128:839–850 25673144 10.1007/s00122-015-2474-4

[CR73] Yu J, Holland JB, McMullen MD, Buckler ES (2008) Genetic design and statistical power of nested association mapping in maize. Genetics 178(1):539–551 18202393 10.1534/genetics.107.074245 PMC2206100

[CR74] Zhang C, Dong S, Xu J, He W, Yang T (2019) PopLDdecay: a fast and effective tool for linkage disequilibrium decay analysis based on variant call format files. Bioinformatics 35(10):1786–1788 30321304 10.1093/bioinformatics/bty875

[CR75] Zhang H, Goettel W, Song Q, Jiang H, Hu Z, Wang ML, An YC (2020) Selection of GmSWEET39 for oil and protein improvement in soybean. PLoS Genet 16(11):e1009114 33175845 10.1371/journal.pgen.1009114 PMC7721174

[CR76] Zou M, Shabala S, Zhao C, Zhou M (2024) Molecular mechanisms and regulation of recombination frequency and distribution in plants. Theor Appl Genet 137(4):86 38512498 10.1007/s00122-024-04590-4 PMC10957645

