# Supplementary figures and images for: Characterization of a G. max × G. soja nested association mapping population and identification of loci controlling seed composition traits from wild soybean
Source: Theor Appl Genet. 2025 Mar 7;138(3):65. doi: 10.1007/s00122-025-04848-5 (PMC11889062; doi:10.1007/s00122-025-04848-5)

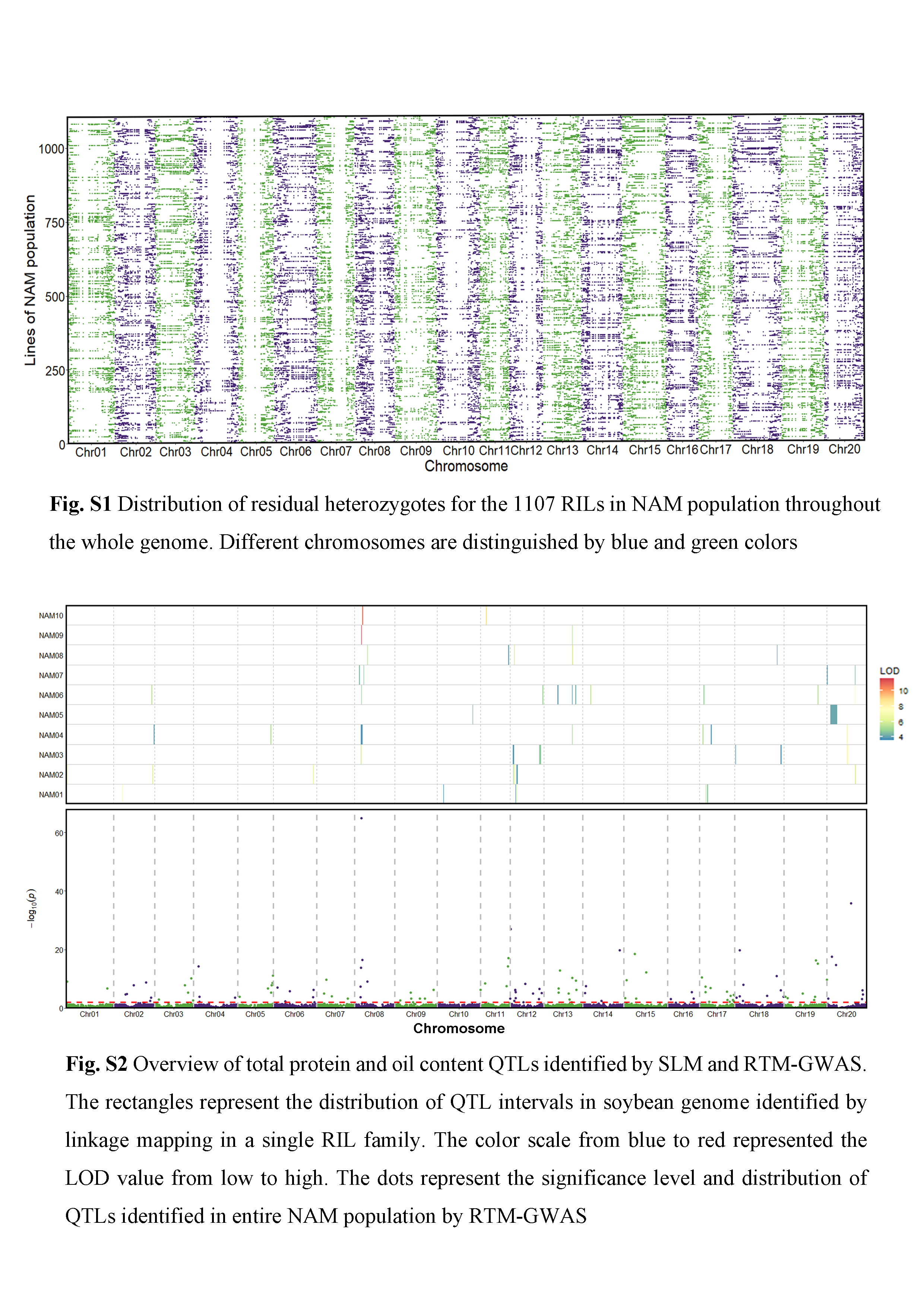

Supplement: Supplementary file 1 — Supplementary file1 (TIF 6536 KB) [file 122_2025_4848_MOESM1_ESM.tif]

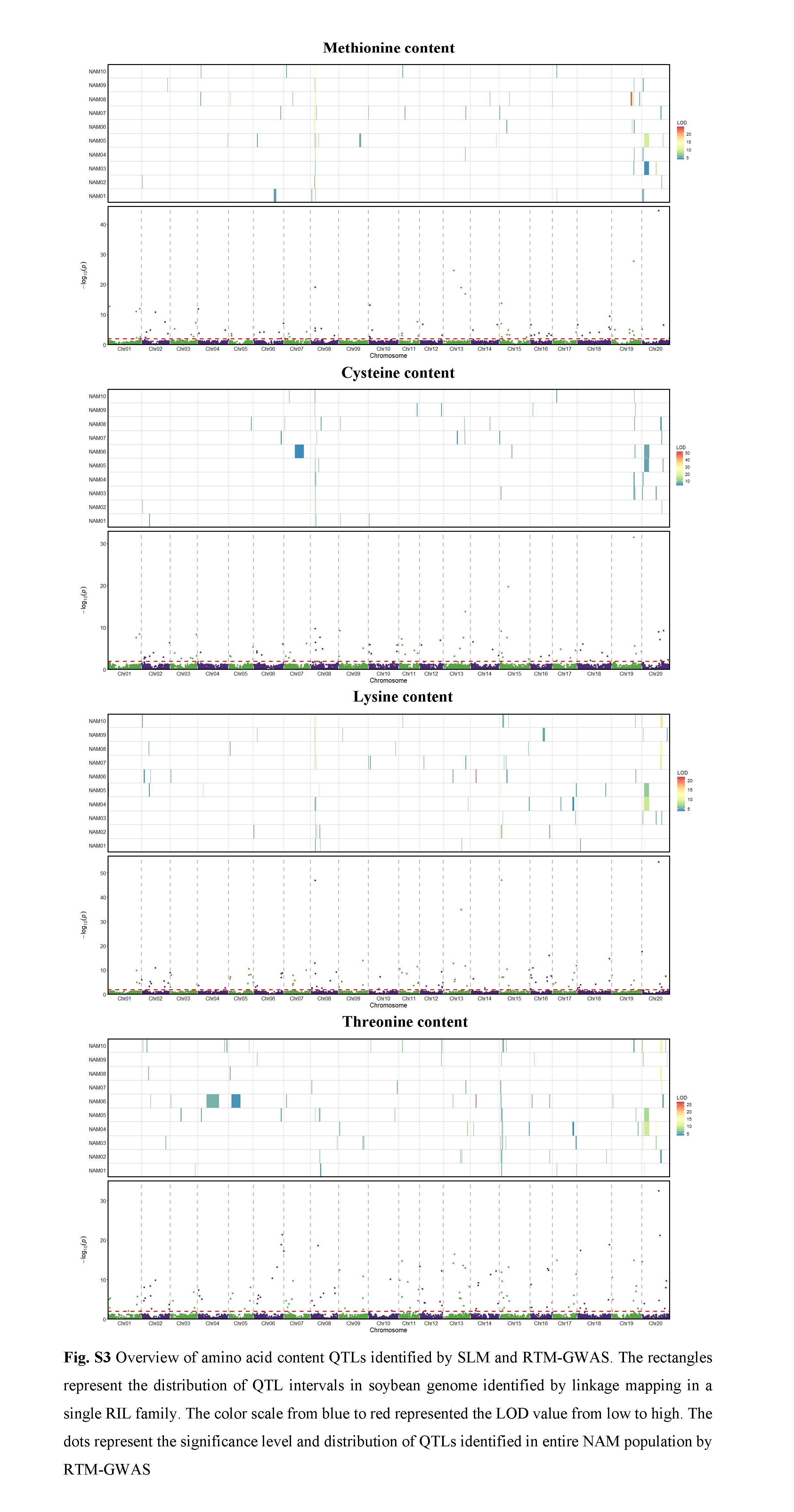

Supplement: Supplementary file 2 — Supplementary file2 (TIF 1045 KB) [file 122_2025_4848_MOESM2_ESM.tif]

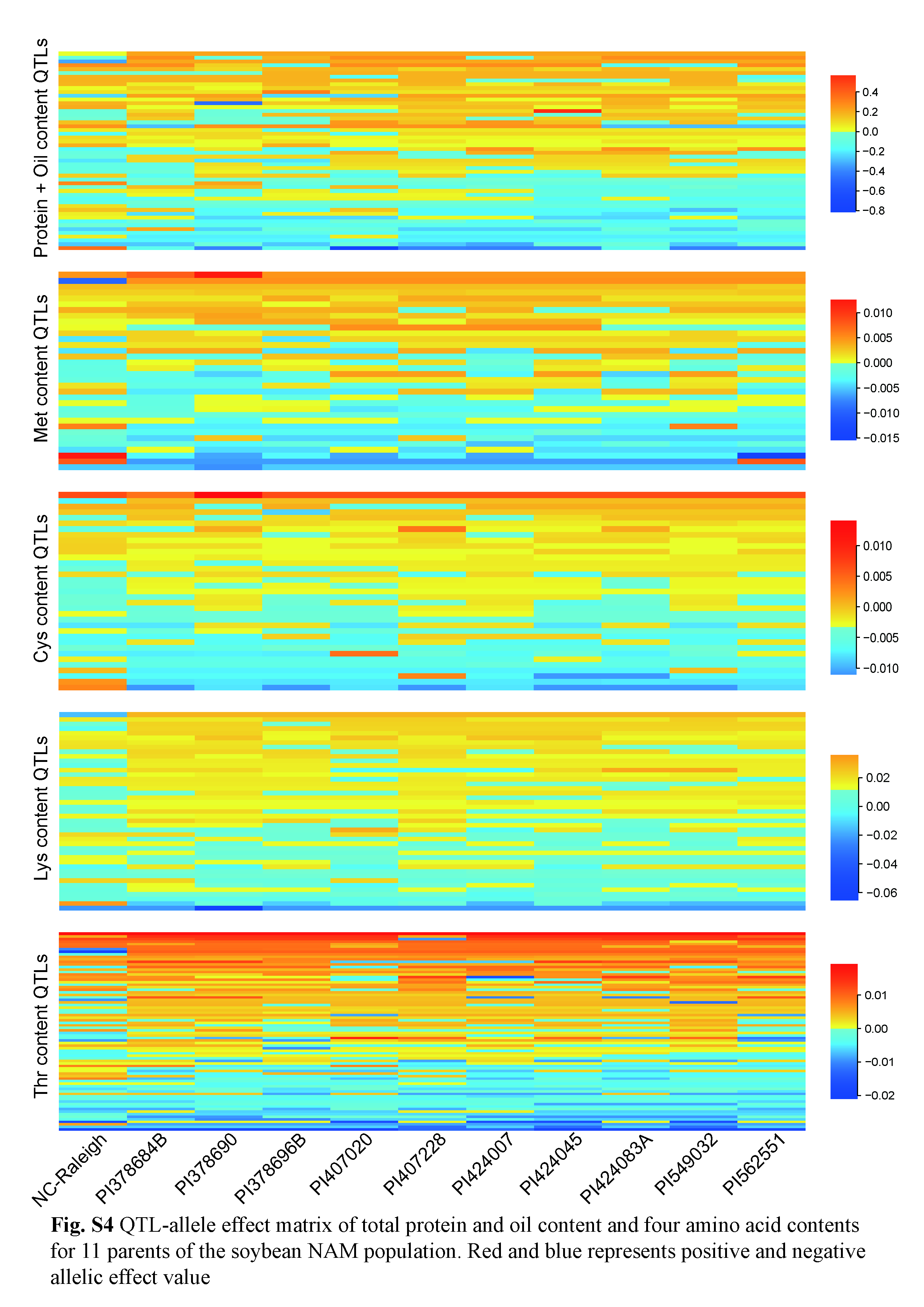

Supplement: Supplementary file 3 — Supplementary file3 (TIF 1763 KB) [file 122_2025_4848_MOESM3_ESM.tif]

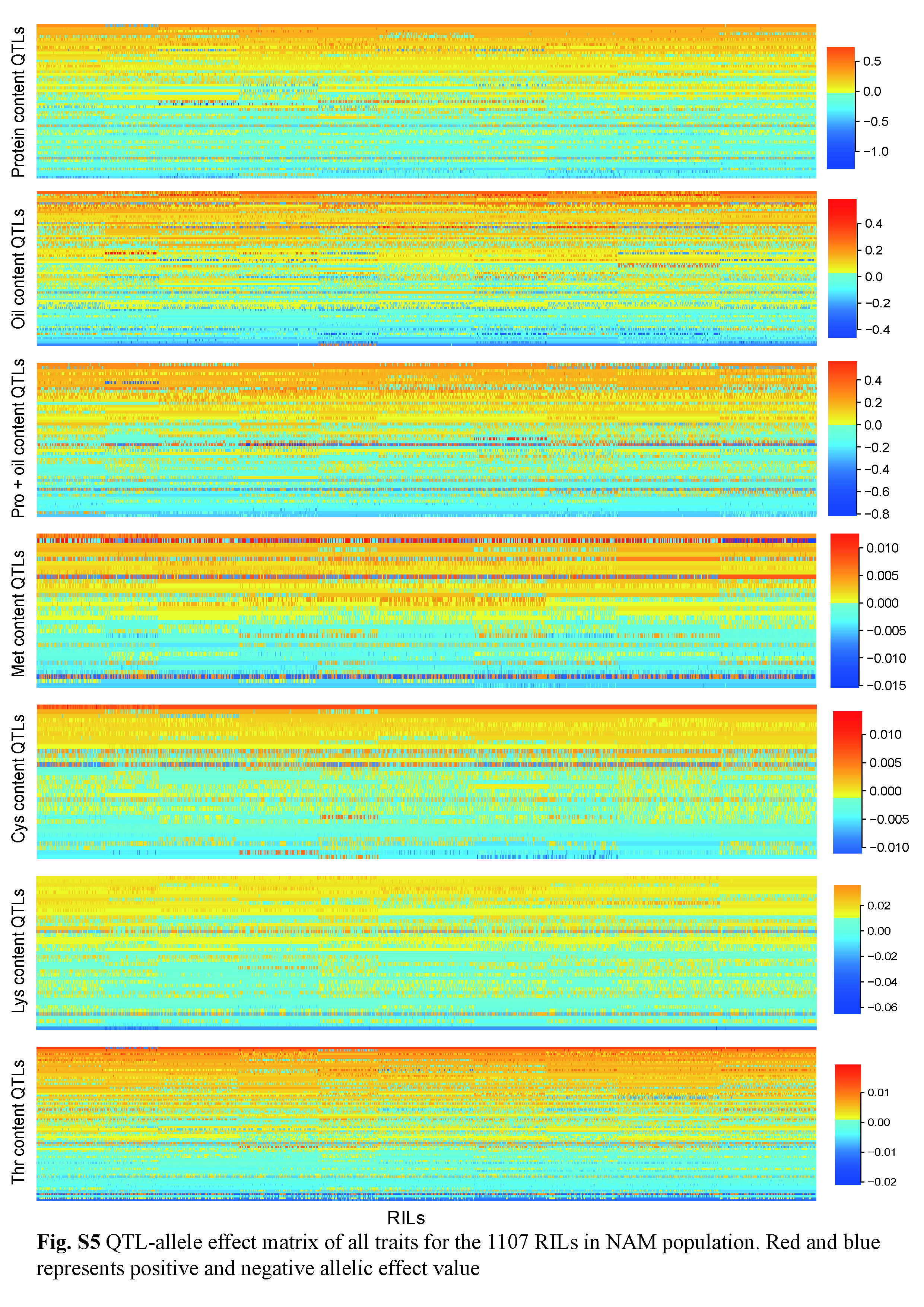

Supplement: Supplementary file 4 — Supplementary file4 (TIF 9500 KB) [file 122_2025_4848_MOESM4_ESM.tif]

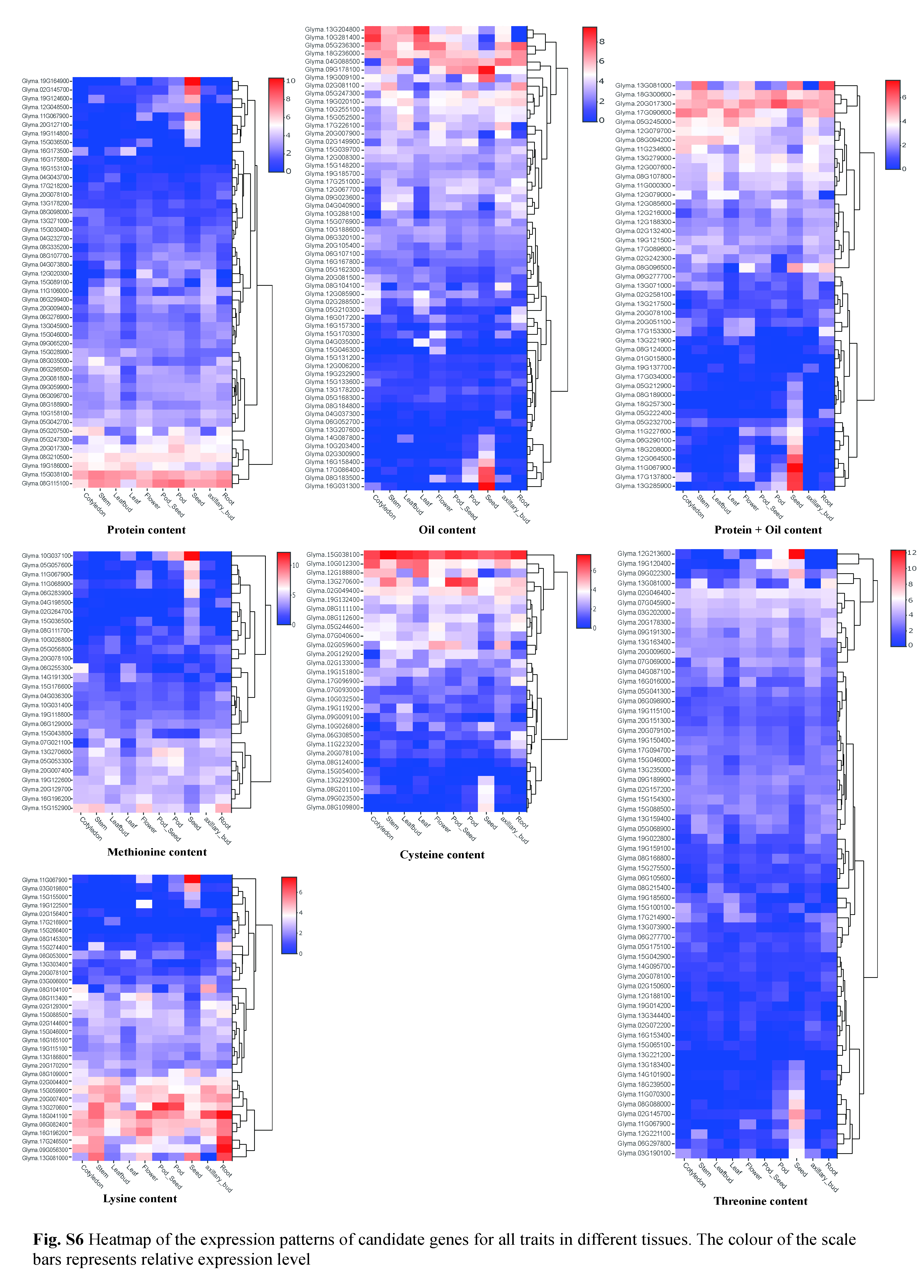

Supplement: Supplementary file 5 — Supplementary file5 (TIF 6544 KB) [file 122_2025_4848_MOESM5_ESM.tif]
